# Revisiting the Growth Modulon of *Corynebacterium glutamicum* Under Glucose Limited Chemostat Conditions

**DOI:** 10.3389/fbioe.2020.584614

**Published:** 2020-10-15

**Authors:** Michaela Graf, Thorsten Haas, Attila Teleki, André Feith, Martin Cerff, Wolfgang Wiechert, Katharina Nöh, Tobias Busche, Jörn Kalinowski, Ralf Takors

**Affiliations:** ^1^Institute of Biochemical Engineering, University of Stuttgart, Stuttgart, Germany; ^2^Institute of Bio- and Geosciences, IBG-1: Biotechnology, Forschungszentrum Jülich GmbH, Jülich, Germany; ^3^Center for Biotechnology, Bielefeld University, Bielefeld, Germany; ^4^Institute for Biology-Microbiology, Freie Universität Berlin, Berlin, Germany

**Keywords:** *Corynebacterium glutamicum*, growth rate, metabolome, transcriptome, continuous bioprocess, stationary flux analysis

## Abstract

Increasing the growth rate of the industrial host *Corynebacterium glutamicum* is a promising target to rise productivities of growth coupled product formation. As a prerequisite, detailed knowledge about the tight regulation network is necessary for identifying promising metabolic engineering goals. Here, we present comprehensive metabolic and transcriptional analysis of *C. glutamicum* ATCC 13032 growing under glucose limited chemostat conditions with μ = 0.2, 0.3, and 0.4 h^–1^. Intermediates of central metabolism mostly showed rising pool sizes with increasing growth. ^13^C-metabolic flux analysis (^13^C-MFA) underlined the fundamental role of central metabolism for the supply of precursors, redox, and energy equivalents. Global, growth-associated, concerted transcriptional patterns were not detected giving rise to the conclusion that glycolysis, pentose-phosphate pathway, and citric acid cycle are predominately metabolically controlled under glucose-limiting chemostat conditions. However, evidence is found that transcriptional regulation takes control over glycolysis once glucose-rich growth conditions are installed.

## Introduction

*Corynebacterium glutamicum*, discovered in 1957 as a soil bacterium, has gained prominent acceptance in industry for the production of amino acids, organic acids, and even proteins (Hermann, 2003; Wendisch et al., 2006; Freudl, 2017). Beside the broad availability of fine-tuned tools to genetically engineer the strain, *C. glutamicum* offers intrinsic features which are of high interest for use in industrial production. In particular, the robustness for large-scale application is one of its outstanding traits (Vertès et al., 2012).

However, a putative drawback lies in the somewhat reduced growth rates compared to other hosts such as *Escherichia coli*. With about 0.4 h^–1^ wild-type (WT) *C. glutamicum* shows only moderate growth in synthetic media in classical batch cultivations (Blombach et al., 2013; Grünberger et al., 2013; Graf et al., 2018; Haas et al., 2019) compared to about 0.7–0.8 h^–1^ maximum growth rate of other prokaryotes such as *E. coli*, 1.7 h^–1^ for the putative future competitors *Vibrio natriegens* (Hoffart et al., 2017) and *Geobacillus* LC300 with 2.15 h^–1^ (Long et al., 2017). Relatively low space-time yields compared to other hosts may be the consequence. Therefore, several authors have tried to boost the growth of *C. glutamicum* applying growth evolution studies aiming for a “natural” selection of a faster growing *C. glutamicum* strain (Pfeifer et al., 2017; Wang et al., 2018; Graf et al., 2019). As a result, several gene mutations were identified serving as putative targets for metabolic engineering toward higher growth under controlled conditions.

Besides these efforts to accelerate the growth rate, other studies aimed to at understand growth regulation of *C. glutamicum* deciphering potential limitations to improve growth coupled productivities (Grünberger et al., 2013; Unthan et al., 2014). More precisely, the cell’s intrinsic regulation was analyzed by employing tools from the systems biology toolbox, i.e., metabolomics, transcriptomics, fluxomics, and proteomics. An essential prerequisite for these “omics” disciplines is the establishment of highly reproducible and stable experimental cultivation environments (Hoskisson and Hobbs, 2005; Bull, 2010). Therefore, continuous bioprocess setups are commonly used since they make it possible to grow a cell population in a metabolic steady state by limiting one growth substrate, e.g., the carbon source. Manipulation of the dilution rate translates to an adjustment of limiting substrate supply whereby the growth rate of the investigated strain can be ultimately set to a precise rate. With regard to *C. glutamicum* WT ATCC 17965, continuous chemostat processes were performed by Cocaign-Bousquet et al. (1996) to examine growth kinetics and glucose consumption at μ = 0.1–0.55 h^–1^. Their results hinted at the presence of a secondary transport system for glucose beside the phosphotranferase system (PTS) which was confirmed by two different groups later (Ikeda et al., 2011; Lindner et al., 2011). Silberbach et al. (2005) were among the first to perform systems biology analysis on *C. glutamicum* ATCC 13032 by analyzing the cell’s transcriptional and proteomic response to ammonium limitation at two different growth rates. They identified growth rate dependent expression of ribosomal proteins and of F_0_F_1_ subunits of ATP-synthase. Another study with a systems biology viewpoint was recently performed by Noack et al. (2017) conducting chemostat cultivations to record the proteomic response of *C. glutamicum* WT ATCC 13032 within a broad range of growth rates (μ = 0.05–0.35 h^–1^). Under the tested glucose-limited growth conditions, Noack et al. (2017) concluded that metabolic fluxes within *C. glutamicum*’s central carbon metabolism are likely more affected by concentrations of local substrates and allosteric metabolite effectors than by the concentration of enzymes. Recently, another systems biology study was performed in traditional non-substrate limited batch cultivations to investigate determinants of global regulation orchestrating the growth of *C. glutamicum* (Haas et al., 2019). The so-called growth modulon was discovered by investigating transcriptional dynamics of strains in batch cultivation. Growth control was observed to be well distributed within a tightly networked system of transcriptional regulators controlling different branches of central metabolism.

Based on these recent systems biology insights, the current study tries to broaden knowledge about the strain’s metabolic, transcriptional, and fluxomic response to different growth rates. Accordingly, we continuously cultivated *C. glutamicum* under glucose-limited growth conditions setting dilution rates of 0.2, 0.3, and 0.4 h^–1^, applying the same strain as Haas et al. (2019). We analyzed whether the transcriptional regulatory response identified in glucose-rich batch cultures is similar to the one operational under chemostat conditions at similar growth rates. Thereof we investigated how comparable transcriptional data of batch and continuous culture are. In other words we wonder: How similar are regulatory patterns of *C. glutamicum* growing with equal rates but under different nutrient availability?

## Materials and Methods

### Bacterial Strain and Pre-culture Cultivation

All cultivations of this study were performed with the WT strain *C. glutamicum* ATCC 13032 obtained from the American Type Culture Collection (ATCC, Manassas, VA, United States). Cells from a glycerol stock working cell bank were spread on 2 × tryptone-yeast extract (2 × TY) medium (Sambrook, 2001) agar plates and incubated for 48–60 h at 30°C. Thereof, extracted colonies served to inoculate 5 mL sterile 2 × TY medium in glass reaction tubes and were incubated for 8 h at 30°C on a bench-top rotary shaker (Infors HT, Bottmingen, Switzerland) at 120 rpm. Subsequently, individual shaking flasks filled with 50 mL of sterile CGXII minimal medium (Grünberger et al., 2013) supplemented with 40 g L^–1^ glucose were inoculated with the pre-culture content of one glass reaction tube, respectively. Shaking flask pre-cultures were incubated overnight at 30°C and 120 rpm.

### Continuous Bioreactor Cultivations

#### Bioreactor Setup

Continuous chemostat processes with *C. glutamicum* were conducted in a 3 L steel bioreactor (KLF 2000, Bioengineering, Switzerland) equipped with process control (Lab view 2010, National Labs). Cooling and heating elements and a temperature sensor (Pt100, Bioengineering, Switzerland) ensured constant cultivation temperature of 30°C. Mixing was realized with a six blade Rushton turbine. A pressure probe (PR-35 X HT, KELLER Druckmesstechnik, Jestetten, Germany) and an automatic exhaust valve were installed at the head of the reactor to regulate pressure (1.5 bar). For aeration, compressed air was channeled through a mass flow controller (MFC GFC171S, Analyt-MTC GmbH, Müllheim, Germany) and filtered aseptically (Midisart^®^ 2000, Sartorius Stedim Biotech, Goettingen, Germany) before entering the bioreactor. Dissolved oxygen was monitored by an amperometric pO_2_ electrode (InPro 6800, Mettler-Toledo, Germany) and pH with a conventional pH probe (405-DPAS-SC-K8S, Mettler-Toledo, Germany). Molar fractions of oxygen and carbon dioxide in the exhaust gas were measured by a sensor (BCP-O2/CO2, BlueSens, Germany).

To enable continuous processes in chemostat mode at fixed dilution rates (*D*, h^–1^) equaling the growth rate (μ, h^–1^; see section “Determination of Kinetic Parameters”), the advanced controller scheme described in Graf et al. (2019) was employed. Thereby, a defined reaction volume in the bioreactor was maintained and constant feed and harvest rates were ensured. Prior to all cultivations, the reactor was sterilized with 1 mol L^–1^ K_2_HPO_4_/KH_2_HPO_4_ buffer (pH 7) for 20 min at 121°C. Prior removal of the buffer, the drain of the reactor was sterilized *in situ* with pure steam for 10 min.

#### Chemostat Processes at μ = 0.2, 0.3, 0.4 h^–1^

All chemostat processes started with an initial batch cultivation. Therefore, sterile CGXII minimal medium (Grünberger et al., 2013) supplemented with 12 g D-glucose L^–1^ was pumped from a stirred sterile medium feed reservoir (later employed for continuous mode) into the sterile bioreactor until a volume of 1.4 L was reached. The required amount of *C. glutamicum* biomass from pre-culture shaking flaks (see section “Bacterial Strain and Pre-culture Cultivation”) was harvested and resuspended in 100 mL 9 g NaCl L^–1^ solution yielding an inoculation biomass density of 0.3 g_CDW_ L^–1^ and a cultivation volume of 1.5 L. During batch and continuous cultivation, pH was controlled at 7.4 by automatically adding 25% (v/v) NH_4_OH solution. In batch mode, pO_2_ was maintained above 30% by 50 rpm stepwise increase of the impeller speed (initial: 250 rpm) and analogous rise of aeration rate by 0.15 L min^–1^ (initial: 0.15 L min^–1^). Antifoam agent (Struktol^®^ J 647, Schill + Seilacher, Hamburg, Germany) was manually added when necessary. After about 7.5 h of exponential growth, the sharp increase of the pO_2_ signal indicated glucose depletion.

Thereupon, the continuous mode was initiated by starting the control regime (Graf et al., 2019), adding antifoam agent constantly and fixing impeller speed and aeration rate to 700 rpm and 1 L min^–1^, respectively. During the course of one continuous experiment, dilution rates of *D* = 0.2, 0.3, and 0.4 h^–1^ were sequentially installed. Constant antifoam concentrations were achieved by increasing related feed rates proportionally (80, 120, 160 μL h^–1^). Sampling of each growth rate was performed after at least five residence times (RTs), i.e., 25, 16.67, 12.5 h, respectively. Accordingly, the achievement of a metabolic steady state of the culture was ensured (Zamboni et al., 2009). Sampling comprised withdrawing biosuspension for biomass determination [optical density (OD_600_) and cell dry weight (CDW) measurements], providing cell-free filtrates for extracellular metabolite analyses, and determining total inorganic carbon (TIC) and organic carbon (TOC) as described in the Supplementary Material section “Analysis of Exometabolome.” Moreover, intracellular metabolome and transcriptome samples were taken and analyzed as described in the same Supplementary Material sections “Sampling, Quenching, and Extraction for Absolute Quantification of Intracellular Pool Concentrations” and “Flux Estimation,” respectively. Non-labeled intracellular pools were measured by HILIC-based QQQ-MS/MS analysis (see Supplementary Material section “LC-MS-Based Quantification of Intracellular Pool Concentrations and ^13^C-Labeling Dynamics”) and absolutely quantified by isotope dilution mass spectrometry (IDMS) with a constant addition of (U-^13^C)-labeled *C. glutamicum* extracts (see Supplementary Material section “Generation of Fully ^13^C-Labeled *C. glutamicum* extracts for IDMS”). Apart from transcriptome samples, conventional and metabolome samples were withdrawn 3× in 1.5 h intervals at each growth rate. Continuous chemostat processes at *D* = 0.2, 0.3, and 0.4 h^–1^ were conducted in biological triplicates following the described scheme.

#### ^13^C-Labeling Experiment

The ^13^C-carbon labeling experiment (CLE) in chemostat mode (*D* = 0.4 h^–1^) was initiated with a batch cultivation as described in section “Chemostat Processes at μ = 0.2, 0.3, 0.4 h^–1^,” but aiming for a working volume of 1.2 L instead of 1.5 L. Therefore, 1.1 L sterile CGXII minimal medium (Grünberger et al., 2013) supplemented with 12 g D-glucose L^–1^ was retrieved from the sterile feed reservoir and stirrer speed and aeration were set to 600 rpm and 0.8 L min^–1^, respectively.

Unlimited batch growth prevailed for 7.45 h (μ = 0.42 h^–1^) until glucose was depleted from the medium. The continuous mode was started by feeding fresh supplemented CGXII medium with a dilution rate of 0.4 h^–1^ while keeping the working volume constant (1.2 L) and constantly feeding 200 μL h^–1^ antifoam agent to prevent excessive foaming. After 12.5 h equaling five RTs, online measurements of O_2_ and CO_2_ in the exhaust gas indicated a metabolic steady state of the culture. Conventional sampling was performed as described in Supplementary Material section “Analysis of Exometabolome.” After five more RTs (12.5 h), the CGXII medium feed containing non-labeled (U-^12^C)-D-glucose was switched to labeled CGXII feed containing 67% (U-^13^C)-D-glucose and 33% (1-^13^C)-D-glucose according to the previously performed tracer design (see section “*A priori* Tracer Design”). After four RTs (10 h), samples for intracellular isotopologue metabolome analyses were withdrawn (see Supplementary Material section “Sampling, Quenching, and Extraction for Quantification of ^13^C-Isotopic Labeling Dynamics”) as a stationary isotopic enrichment in *C. glutamicum*’s intracellular metabolites was expected at this time. Isotopic distributions of ^13^C-labeled and non-labeled intracellular pools of analog samples were quantified by HILIC-based QTOF-HRMS analysis (see Supplementary Material section “LC-MS-Based Quantification of Intracellular Pool Concentrations and ^13^C-Labeling Dynamics”). For technical replicates of this measurement, intracellular metabolome samples were withdrawn 30, 20, and 10 min before reaching four RTs, and 10 min afterward.

### Transcriptome Analysis

Transcriptome sampling was performed by withdrawing 1 mL biosuspension from the bioreactor, centrifuging at 20,817 × *g* and 4°C for 30 s (5430 R, Eppendorf, Hamburg, Germany), discarding the supernatant and immediately quenching the biomass pellet in liquid nitrogen. Extraction and isolation of RNA from the samples, whole transcriptome sequencing, read mapping, and raw read count calculation was performed as described in Haas et al. (2019). The raw sequencing data have been deposited in the ArrayExpress database at EMBL-EBI under accession number E-MTAB-9371. Transcript data analysis was performed using R version 3.5.0. Raw read counts were normalized using the Relative Log Expression function of the DESeq2 (Love et al., 2014) package. Differential gene expression was determined using the maSigPro (Nueda et al., 2014) package in “generalized linear model” mode. Clustering of the genes according to expression trend was performed through hclust as implemented in maSigPro.

### ^13^C-Metabolic Flux Analysis: Modeling and Computational Procedures

#### Metabolic Network Model for ^13^C-MFA

The metabolic network model of *C. glutamicum* WT was taken from Kappelmann et al. (2016) and slightly modified for experimental design and flux fitting (see Supplementary Material section “^13^C-MFA”). The models were composed according to the workflow described in Nöh et al. (2015) and formulated in FluxML format (Beyß et al., 2019). The models contain the major pathways of central carbon metabolism such as glycolysis, pentose phosphate pathway (PPP), anaplerotic reactions, tricarboxylic acid (TCA) cycle, and all pathways for amino acid synthesis. In addition, protocatechuic acid (PCA) oxidation into the TCA cycle was taken into account (Unthan et al., 2014). A lumped biomass equation was formulated representing the cellular composition (Eggeling and Bott(eds), 2005). Transport reactions were considered for glucose, PCA and carbon dioxide (CO_2_). Carbon atom transitions were formulated for each reaction. For reactions with C-symmetric reactants (in the TCA cycle and lysine synthesis), scrambling reactions were introduced and for each variant equal fluxes assumed. The metabolic model used for flux fitting comprises 45 balanced intracellular and 4 non-balanced extracellular metabolite pools, connected through 87 metabolic reactions (22 bidirectional and 65 unidirectional). The model has 27 degrees of freedom (7 net and 20 exchange fluxes), whereas the network model used for tracer design was slightly simpler (22 free parameters, 5 net fluxes, and 17 exchange fluxes). Full model specifications are provided in the Supplementary Data FluxML Files.

#### *A priori* Tracer Design

To determine an informative, yet cost efficient tracer mixture for the CLE at *D* = 0.4 h^–1^, an experimental design study was performed using the high-performance simulation framework 13CFLUX2 (Möllney et al., 1999; Weitzel et al., 2013). To this end, a measurement configuration was formulated on the basis of a previous ^13^C-tracer quantification study (Feith et al., 2019), and six glucose tracers were considered [(U-^12^C)-, (U-^13^C)-, (1-^13^C)-, (1,2-^13^C)-, (6-^13^C)-, and (5,6-^13^C)-D-glucose]. The model used for the design as well as the assumed measured fragments and rates including their corresponding expected errors are detailed in the Supplementary Material section “Model Setup.”

#### External Rate Estimation

For the CLE at *D* = 0.4 h^–1^, extracellular biomass-specific rates (growth rate μ, glucose consumption rate *r*_GLC_, CO_2_-production rate *r_CO__2_*, PCA uptake rate *r*_PCA_) were estimated using an ODE-based bioprocess model adapted from Cerff et al. (2013) (see details in Supplementary Material section “Bioprocess Model Used for External Rate Estimation”). Model parameters were estimated using weighted least squares regression. Standard deviations of model parameters were estimated using Monte-Carlo simulations (1,000 runs with random initial parameter values). Estimates of biomass-specific rates including their errors were used for ^13^C-metabolic flux analysis (^13^C-MFA). The bioprocess model was implemented in Matlab (Mathworks, Natick, MA, United States). Monte Carlo simulations were performed with custom Matlab scripts.

#### Intracellular Flux Estimation

After measurements became available, metabolic fluxes were estimated by minimizing the variance-weighted sum of squared residual (SSR) between the simulated quantities and the measurements, i.e., the extracellular biomass specific rates (Supplementary Table 3) and mass isotopomer distributions (MIDs; see Supplementary Material section “Flux Estimation”). Prior to ^13^C-MFA, raw MIDs were corrected for natural isotope abundance using the ICT-toolbox (Jungreuthmayer et al., 2016). Corrected MIDs were derived for the sample taken at *t* = 10.17 h after introduction of ^13^C-label. MID standard deviations were set to 2.5 mol%, based on analytical experience and supported by the analysis of four samples drawn in the isotopic quasi-stationary regime (9.67–10.17 h after introduction of ^13^C-tracer). In total 170 measurements (164 labeling data for 28 metabolites and 6 biomass-specific extracellular rates) were used to determine 55 model parameters (27 free fluxes and 28 measurement group scale factors).

Flux estimation was performed using 13CFLUX2 using the optimizer LINCOA (Powell, 2015) and the NAG optimization toolbox (National Algorithms Group, Oxford, United Kingdom). To minimize the risk of suboptimal local regression solutions, a multi-start heuristic was applied combining gradient-free (LINCOA) and gradient-based (NAG) optimizers, which was initialized 1,000 times from random starting flux distributions. The set of fluxes that resulted in the minimum weighted deviation between experimentally observed (MID and extracellular rates) and simulated data was reported as best flux estimate for the *in vivo* flux distribution. Statistical analysis was performed according to the method described in Wiechert et al. (1997), yielding simultaneous flux confidence intervals.

### Determination of Kinetic Parameters

#### Growth Rate

According to the continuous mass balance (cf. Eq. 1 in Supplementary Material section “Bioprocess Model Used for External Rate Estimation”) and under the assumption of metabolic stationarity of the culture, the set dilution rate *D* (in h^–1^) equals the exponential growth rate μ (in h^–1^) of the culture:

μ=D

Metabolic steady states were ensured by employing the controller scheme described in Graf et al. (2019).

#### Biomass Specific Glucose Consumption Rate and Biomass-Glucose Yield

According to the continuous glucose mass balance (cf. Eq. 2 in Supplementary Material section “Bioprocess Model Used for External Rate Estimation”) and under the assumption of metabolic stationarity of the culture, the biomass-specific glucose consumption rate *q*_GLC_ (in g g_CDW_^–1^ h^–1^) is:

$$q_{\text{GLC}}=D\cdot \frac{c_{\mathrm{GLC},\mathrm{feed}}}{c_{X}}$$

where *c*_GLC,feed_ (in g L^–1^) is the glucose concentration of the feed medium and *c*_X_ (in g L^–1^) is the biomass concentration of the culture. Residual glucose in cell free biosuspension samples was below the detection limit and was therefore neglected in the calculation.

Furthermore, the biomass-glucose yield (*Y_X__,GLC_*) was determined by:

$$Y_{X,GLC}=\frac{D}{q_{\text{GLC}}}=\frac{c_{x}}{c_{\mathrm{GLC},\mathrm{feed}}}$$

#### Respiratory Rates

Biomass-specific respiratory rates (*q_O__2_*, *q_CO__2_*) were calculated by dividing volumetric oxygen consumption or carbon dioxide emission rates by the biomass concentration determined at set dilution rate (cf. Eq. 9 in Supplementary Material section “Bioprocess Model Used for External Rate Estimation”). The carbon dioxide emission rate was corrected by TIC-measurements using a total carbon-analyzer (Multi N/C 2100s, Analytik Jena, Jena, Germany).

## Results

### Chemostat Cultivations at μ = 0.2, 0.3, and 0.4 h^–1^

#### Continuous Processes and Kinetic Parameters

**FIGURE 1:**
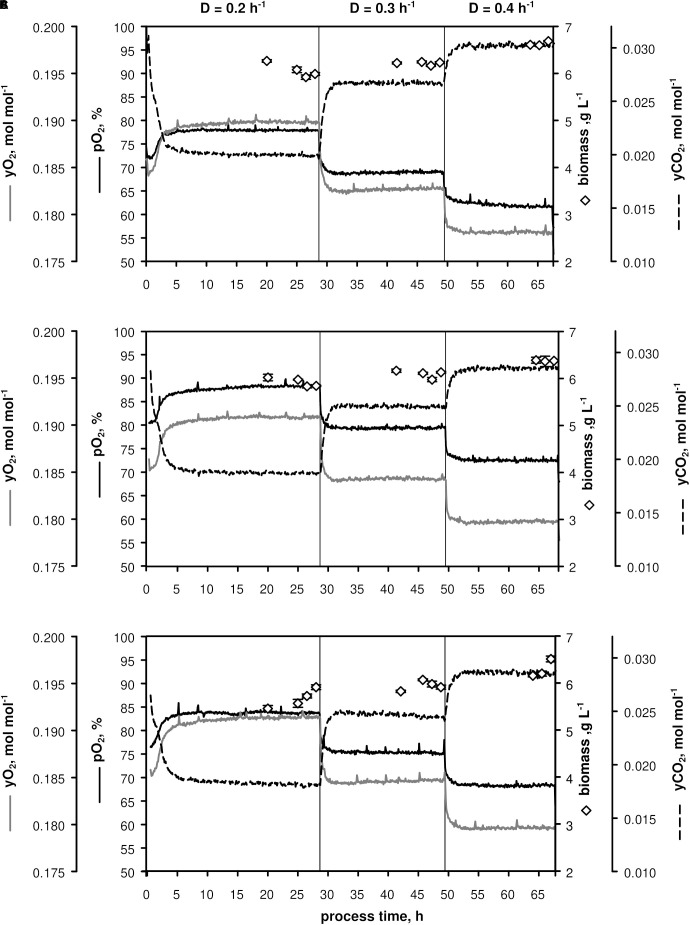
Process parameters of triplicate chemostats **(A–C)** with *C. glutamicum* WT: dissolved oxygen (*pO*_2_), off-gas molar oxygen (*y_O__2_*) and carbon dioxide (*y_CO__2_*), biomass concentration (black diamonds). The dilution rate (*D*) was set to 0.2, 0.3, and 0.4 h^– 1^ and sampling was performed after five residence times. Stirrer speed and aeration remained at 700 min^– 1^ and 0.67 vvm, respectively. Depicted standard deviations of biomass measurements were calculated from three technical replicates per sample.

Three biological replicates (A–C, Figure 1) were conducted in chemostat mode employing CGXII minimal medium (Grünberger et al., 2013) supplemented with 12 g D-glucose L^–1^. After about 7.5 h batch phase [μ_max_ = (0.44 ± 0.01) h^–1^] the continuous mode was started with a dilution rate *D* = 0.2 h^–1^ (process time = 0), which was increased to 0.3 h^–1^ after ca. 28 h and to *D* = 0.4 h^–1^ after ca. 49 h. Three independent samples for OD, CDW, filtrate TC/TIC, metabolome, and transcriptome were withdrawn after five RTs within 1.5 h-intervals to monitor the respective metabolic steady-states at *D* = 0.2, 0.3, and 0.4 h^–1^.

**FIGURE 2:**
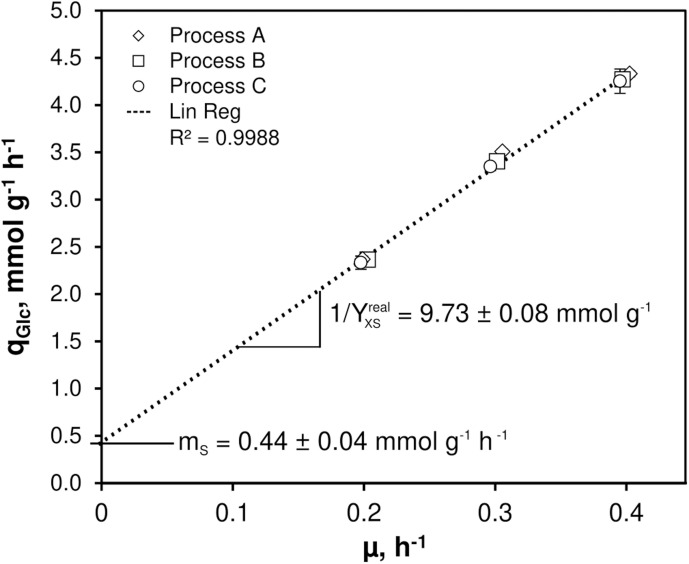
Determination of non-growth rate associated maintenance parameter (m_S_) and growth rate associated maintenance parameter (1/Y_real,xGLC_) for *C. glutamicum* WT. Growth rates (μ) and corresponding glucose consumption rates (q_GLC_) derived from three individual continuous chemostat processes (A, B, C) built the basis for the linear regression revealing m_S_ and 1/Y_real,xGLC_ as indicated. The strain was cultivated in CGXII minimal medium supplemented with 30 mg PCA L^– 1^ and 12 g D-glucose L^– 1^ as carbon source. Mean values ± standard deviations were calculated from three technical replicates per growth rate installed in each process.

As illustrated in Figure 1, only minor differences occurred between the triplicates mirroring slightly varying feed glucose concentrations (*c_G__LC_,_feed_* = 12.47 ± 0.35 g L^–1^). Biomass levels elevated from 5.87 ± 0.13 to 6.43 ± 0.18 g L^–1^ with rising *D*. By analogy, glucose-to-biomass yields rose from 0.47 ± 0.01 to 0.52 ± 0.00 g g_CDW_^–1^ (Table 1) which caused partially growth coupled changes of *q_G__LC_*, *q_O__2_*, and *q_CO__2_*. Respiratory quotients (RQ) remained at 1. All carbon balances closed well with 99 ± 1, 98 ± 2, and 96 ± 3% for the growth rates μ = 0.2, 0.3, and 0.4 h^–1^, respectively (data not shown). No residual glucose or *C. glutamicum* typical by-products such as acetate or pyruvate (Yokota and Lindley, 2005; Han et al., 2008a), lactate or alanine (Blombach et al., 2013) were detectable in biomass-free filtrates analyzed with non-targeted HILIC-QTOF-HRMS (Feith et al., 2019). The growth decoupled maintenance parameters *m*_S_ = (0.44 ± 0.04) mmol D-glucose g_CDW_^–1^ h^–1^ and the growth coupled real glucose-to-biomass yield 1/Y_X__GLC__,real_ = 9.73 ± 0.08 mmol g_CDW_^–1^ (Pirt, 1982) are illustrated in Figure 2.

**TABLE 1:** Listing of kinetic process parameters ± standard deviation calculated from three individual chemostat processes (A, B, C; Figure 1) with *C. glutamicum* cultivated in CGXII medium supplemented with (12.47 ± 0.35) g D-glucose L^–1^.

D, h^–1^	c_X_, g L^–1^	q_Glc_, g g^–1^ h^–1^	Y_XS_, g g^–1^	q_O__2_, mmol g^–1^ h^–1^	q_CO__2_, mmol g^–1^ h^–1^
0.20 ± 0.01	5.87 ± 0.13	0.42 ± 0.01	0.47 ± 0.01	5.36 ± 0.10	5.64 ± 0.14
0.30 ± 0.01	6.09 ± 0.12	0.62 ± 0.01	0.49 ± 0.01	7.16 ± 0.14	7.30 ± 0.18
0.40 ± 0.01	6.43 ± 0.18	0.77 ± 0.01	0.52 ± 0.01	8.15 ± 0.15	7.94 ± 0.10

*c_x_, biomass concentration; q_GLC_, biomass specific glucose uptake rate; Y_X,GLC_, biomass-glucose-yield; q_O2_, molar biomass specific oxygen uptake rate; q_CO2_, carbon dioxide evolution rate.*

### Metabolomics

**FIGURE 3:**
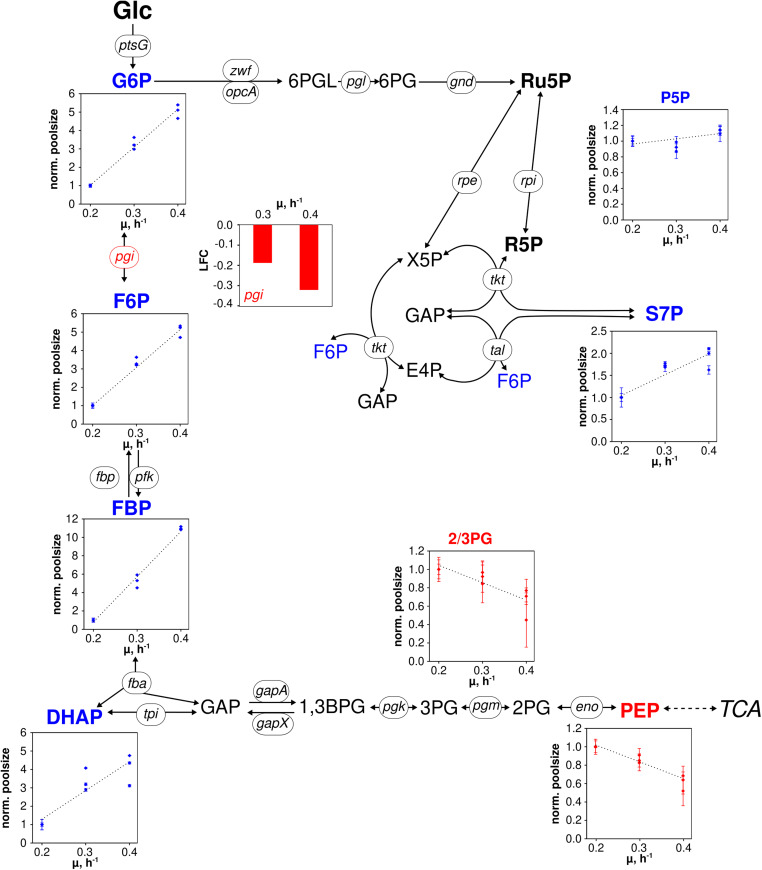
Metabolic and transcriptional response in glycolysis and pentose phosphate pathway of *C. glutamicum* WT to different growth rates (μ = 0.2, 0.3, 0.4 h^– 1^) installed in three independent chemostat processes. Depicted intracellular metabolite concentrations were normalized to respective values determined at μ = 0.2 h^– 1^; values represent the mean of three technical replicates ± standard deviation. Gene expression was normalized to the expression level determined at μ = 0.2 h^– 1^ and is depicted as log fold change (LFC); genes are shown in italic writing. Color code: red, decreasing concentration/expression; blue, increasing concentration/expression.

**FIGURE 4:**
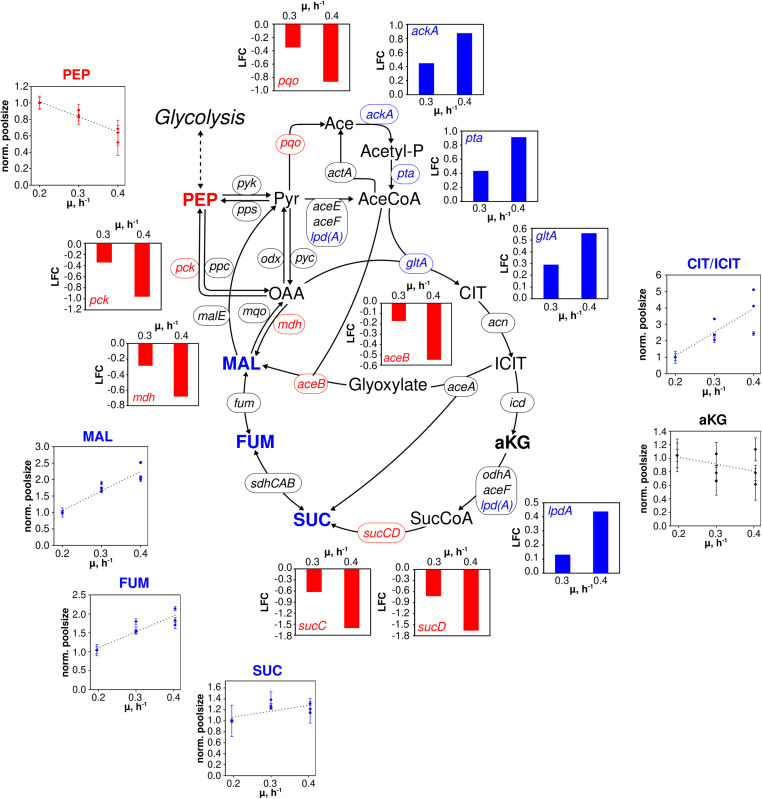
Metabolic and transcriptional response in the TCA cycle of *C. glutamicum* WT to different growth rates (μ = 0.2, 0.3, 0.4 h^– 1^) installed in three independent chemostat processes. Depicted intracellular metabolite concentrations were normalized to respective values determined at μ = 0.2 h^– 1^; values represent the mean of three technical replicates ± standard deviation. Gene expression was normalized to the expression level determined at μ = 0.2 h^– 1^ and is depicted as log fold change (LFC); genes are shown in italic writing. Color code: red, decreasing concentration/expression; blue, increasing concentration/expression.

To account for slightly increasing biomass concentrations, the volumes of extraction solution were adjusted for each sample yielding equal amounts of extracted biomass. Mean values and variances are listed in the Supplementary Material Excel File reflecting the analysis of technical and biological replicates (see Figure 1). As indicated in Table 2, biomass specific pools ranged between 0.01 μmol g_CDW_^–1^ [L-asparagin (ASN)] and 250 μmol g_CDW_^–1^ [L-glutamate (GLU)]. Very good reproducibility of pool sizes was found in biological replicates with low standard errors between 2 and 8%. Few exceptions are the cumulated citrate/isocitrate (CIT/ICIT) and L-proline (PRO) pools revealing standard errors of 13 and 25% at μ = 0.2 h^–1^. Noteworthy, most pool sizes changed proportionally with growth assigning μ = 0.2 h^–1^ either minimum or maximum contents (see Figures 3, 4 and Supplementary Figure 1). The majority of metabolite pools increased with rising μ resulting in 1.2 to 11 times higher concentrations at μ = 0.4 h^–1^ compared to μ = 0.2 h^–1^.

**TABLE 2:** Biomass specific metabolite pool concentrations obtained with the fast-centrifugation treatment and hot-water-extraction from three biological replicates of *C. glutamicum* cultures cultivated in chemostat mode (processes A, B, C) at growth rates of 0.2, 0.3, and 0.4 h^–1^ (Figure 1).

Metabolites	Intracellular pool size, μ mol g_CDW_^–1^		
	μ = 0.2 h^–1^	μ = 0.3 h^–1^	μ = 0.4 h^–1^
G6P	1.32 ± 0.04	4.32 ± 0.16	6.65 ± 0.29
F6P	0.64 ± 0.03	2.16 ± 0.06	3.24 ± 0.08
FBP	0.85 ± 0.07	4.54 ± 0.09	9.33 ± 0.64
DHAP	0.16 ± 0.02	0.52 ± 0.03	0.63 ± 0.04
2/3PG	1.05 ± 0.04	0.96 ± 0.07	0.65 ± 0.02
PEP	1.48 ± 0.04	1.27 ± 0.04	0.90 ± 0.03
CIT/ICIT	0.00 ± 0.00	0.01 ± 0.01	0.01 ± 0.00
aKG	0.75 ± 0.05	0.58 ± 0.01	0.58 ± 0.02
SUC	7.12 ± 0.53	9.27 ± 0.14	8.71 ± 0.52
FUM	0.06 ± 0.00	0.09 ± 0.00	0.11 ± 0.00
MAL	0.34 ± 0.01	0.60 ± 0.01	0.75 ± 0.03
P5P	0.89 ± 0.02	0.82 ± 0.03	1.00 ± 0.04
S7P	0.44 ± 0.03	0.76 ± 0.02	0.83 ± 0.02
ALA	5.19 ± 0.41	5.59 ± 0.23	6.37 ± 0.81
LEU	0.66 ± 0.03	0.64 ± 0.01	0.41 ± 0.01
VAL	2.25 ± 0.01	3.04 ± 0.07	2.63 ± 0.05
ASP	5.44 ± 0.25	4.87 ± 0.20	5.86 ± 0.12
ASN	0.01 ± 0.00	0.01 ± 0.00	0.01 ± 0.00
LYS	0.78 ± 0.06	1.20 ± 0.06	1.45 ± 0.12
THR	0.86 ± 0.02	1.11 ± 0.02	1.35 ± 0.02
ILE	0.50 ± 0.03	0.73 ± 0.02	0.64 ± 0.01
MET	0.61 ± 0.03	0.48 ± 0.01	0.31 ± 0.00
SER	0.99 ± 0.03	1.46 ± 0.04	1.61 ± 0.04
GLY	1.43 ± 0.04	1.98 ± 0.05	3.13 ± 0.06
GLU	255.35 ± 4.06	253.23 ± 5.02	248.58 ± 3.98
GLN	6.22 ± 0.16	6.79 ± 0.14	10.13 ± 0.15
ARG	0.24 ± 0.01	0.25 ± 0.00	0.28 ± 0.00
PRO	0.73 ± 0.19	1.09 ± 0.01	3.89 ± 0.08
TYR	0.03 ± 0.00	0.06 ± 0.00	0.09 ± 0.00
PHE	0.05 ± 0.00	0.08 ± 0.00	0.13 ± 0.00
TRP	0.02 ± 0.00	0.03 ± 0.00	0.03 ± 0.00

*Values represent means ± standard deviations of the biological triplicates.*

To be precise, metabolites of upper glycolysis [glucose 6-phosphate (G6P), fructose 6-phosphate (F6P), fructose 1,6-bisphosphate (FBP), dihydroxyacetone phosphate (DHAP)] linearly increased 5- to 11-fold from μ = 0.2 to 0.4 h^–1^ which is contrasted by intermediates of lower glycolysis [pool of 2-/3-phosphoglyceric acid (2/3PG), phosphoenolpyruvate (PEP)] decreasing about 40% in pool size (Figure 3). In PPP, the summed pool of pentose 5-phosphate sugars (P5P), i.e., ribose 5-phosphate and ribulose 5-phosphate, showed comparably stable pool concentrations (10% decrease at μ = 0.3 h^–1^, 10% increase at μ = 0.4 h^–1^ compared to pool at μ = 0.2 h^–1^), whereas sedoheptulose 7-phosphate (S7P) revealed a steady rise.

In the TCA cycle (Figure 4), remaining trends are observed for alpha ketoglutarate (aKG) and succinate (SUC). In contrast, fumarate (FUM) and L-malate (MAL) showed linear increases between 1.5- and 2-fold over μ. By trend, amino acids, building blocks of protein synthesis, predominately showed rising pool sizes with increasing growth (Supplementary Figure 1). Few exceptions were L-aspartate (ASP), ASN, and GLU which persisted and L-leucine (LEU) and L-methionine (MET) with even decreased concentrations.

#### Transcriptomics

**FIGURE 5:**
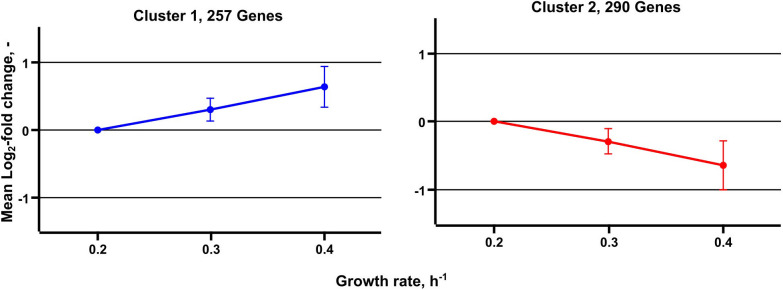
Median expression profiles of differentially expressed genes obtained from transcriptome analysis of *C. glutamicum* cells grown in chemostat cultivation. A total of 257 genes are grouped in cluster 1 and show an increasing trend, 290 genes are grouped in cluster 2 and show a decreasing expression trend.

Metabolic studies were complemented by transcript analysis identifying differentially expressed genes (DEGs) at μ = 0.2, 0.3, and 0.4 h^–1^. Noteworthy, the low growth rate served as reference. In total, 547 DEGs were found grouping in 257 with rising and 290 with decreasing DEGs (Figure 5). By trend, rather proportional correlations with μ were identified.

Differentially expressed genes assigned to genes coding for central metabolism reactions are considered in Figures 3 and 4. Figure 3 comprises reactions of glycolysis and PPP. Notably, only *pgi* was identified as DEG mirroring that expression levels of all other genes persisted despite growth rise from 0.2 to 0.4 h^–1^. In case of *pgi* falling expression levels with increasing growth were detected.

By analogy, of the 28 genes presented in Figure 4 comprising the “extended TCA cycle,” 18 (64%) did not reveal any transcription dynamics with changing growth. Only four were up and six downregulated. Downregulation occurred for genes coding for the formation of MAL, PEP, pyruvate, acetate, and SUC via reducing expression levels of *aceB*, *mdh*, *pck, pqo, sucC, and sucD*, respectively. In particular, *sucC* and *sucD* showed strongest log fold changes (logFCs) with logFC = −1.5 whereas the majority of other DEGs reduction was −0.5 ≤ LFC_max_ ≤ −1. For comparison, upregulation of *ackA*, *pta*, *gltA*, and *lpdA* was less pronounced revealing 0.4 ≤ LFC_max_ ≤ 0.9. Those genes somehow encode the pathway from acetate to citrate.

**FIGURE 6:**
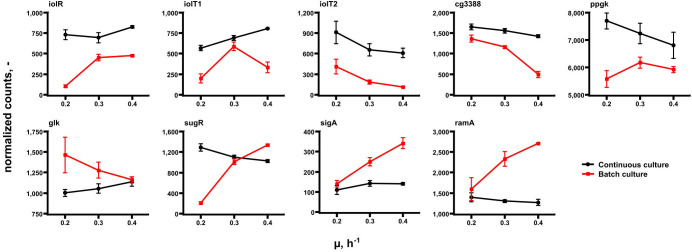
Gene expression levels of a selection of genes coding for glucose uptake and regulation in *C. glutamicum.* Samples were taken at μ = 0.2, 0.3, and 0.4 h^– 1^ in continuous (chemostat) runs (black) and in batch mode (red). Data of the latter are taken from Haas et al. (2019) reflecting the acceleration of growth of the same strain in the same medium after preceding glucose limitation.

Further transcript analysis focused on genes encoding glucose uptake and regulation in *C. glutamicum* (Figure 6). Noteworthy, published data of Haas et al. (2019) serve as reference. The expression profiles mirror transcript dynamics of the same strain in the same synthetic medium after its exposure to glucose limitation (highlighted in red in Figure 6). By trend, *iolT1* (coding for glucose specific EII permease; Ikeda et al., 2011; Lindner et al., 2011) and *glk* (encoding glucokinase; Park et al., 2000) showed rising expression with growth in chemostat mode although *glk* levels in continuous culture were below those of all batch studies. On contrary, *iolT1* expression in chemostat was stronger than in batch cultures. By analogy *iolT2*, the second glucose permease (Ikeda et al., 2011; Lindner et al., 2011), also revealed high expression levels in continuous culture but falling levels with rising growth rate. The same holds true for *ppgK* (polyphosphate glucokinase, Lindner et al., 2010) which phosphorylates glucose to G6P utilizing poly-phosphate pools. *iolR*, *ramA*, cg3388, and *sugR* are putative regulator genes. *iolR* is the known regulator of *iolT1* (Klaffl et al., 2013), whereas cg3388 may exert control on *iolT2* (Brinkrolf et al., 2006). *sugR* represents a transcriptional regulator of sugar metabolism which is likely to be controlled by *ramA*, a “master” regulator in *C. glutamicum* (Cramer and Eikmanns, 2007; Shah et al., 2018). Interestingly, all putative regulators disclosed only moderate expression changes with *ramA* showing the lowest expression level at all.

### ^13^C-Labeling Experiment at μ = 0.4 h^–1^

**FIGURE 7:**
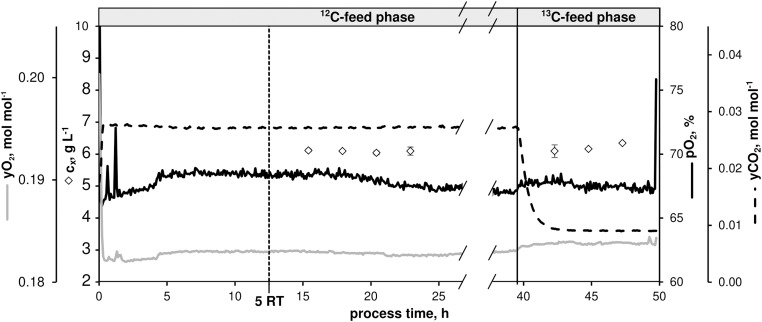
Process parameters of the continuous ^13^C-labeling experiment with *C. glutamicum* WT: dissolved oxygen (*pO*_2_), off-gas molar oxygen (*y_O__2_*) and carbon dioxide (*y_CO__2_*), biomass concentration (black diamonds). The dilution rate (*D*) was set to 0.4 h^– 1^. Sampling in the ^12^C-phase was initiated after five residence times (RT, 12.5 h). After 39.5 h, feed was switched to isotopic CGXII medium containing a 12 g D-glucose L^– 1^ mixture [67% (U-^13^C)-D-glucose and 33% (1-^13^C)-D-glucose]. Residual process parameters were kept constant. Depicted standard deviations of biomass measurements were calculated from three technical replicates per sample.

To further investigate the intracellular flux distribution at μ = 0.4 h^–1^ – the maximum growth rate typically observed under non-limited glucose batch conditions in minimal medium – a CLE was performed in chemostat mode after a non-labeled metabolic steady-state was installed by switching to ^13^C-labeled feed [mixture of 33% (1-^13^C)- and 67% (U-^13^C)-D-glucose, see section “*A priori* Tracer Design”]. Figure 7 illustrates the CLE that ran with the same operating conditions as shown in Figure 1.

After five RTs, non-labeled metabolic steady-state was reached (biomass concentration *c*_X_ = 6.09 ± 0.03 g L^–1^, *pO*_2_ = 67%, *yO*_2_ = 0.18 mol mol^–1^, *yCO*_2_ = 0.03 mol mol^–1^) as evidenced by four fold sampling revealing low standard deviations (Figure 2 and Table 3). A fully closed carbon balance was measured for this phase of the experiment (no data shown).

**TABLE 3:** Kinetic process parameters from the ^13^C-labeling experiment with *C. glutamicum* (Figure 7) using CGXII minimal medium supplemented with 12 g D-glucose L^–1^ [^13^C-phase: 67% (U-^13^C)-D-glucose and 33% (1-^13^C)-D-glucose] at a dilution rate of 0.4 h^–1^.

Phase	*q_G__LC_*, g g_CDW_^–1^ h^–1^	*q_O__2_*, mmol g_CDW_^–1^ h^–1^	*q_CO__2_*, mmol g_CDW_^–1^ h^–1^
^12^C-feed	0.74 ± 0.01	9.26 ± 0.09	9.86 ± 0.10
^13^C-feed	0.77 ± 0.02	9.77 ± 0.14	n.a.

*Listed results are the means of four (^12^C-phase) or three (^13^C-phase) technical replicates. q_GLC_, biomass specific glucose uptake rate; Y_X,GLC_, biomass-glucose-yield; q_O2_, molar biomass specific oxygen uptake rate; q_CO2_, carbon dioxide evolution rate; n.a., not available.*

As indicated in Figure 7, ^13^C-labeled feeding started after 39.5 h causing dropping *yCO*_2_ signals as a well-known interference between newly produced isotopic CO_2_ and near-infrared exhaust gas measurements. Because of slightly higher glucose feed concentrations in the labeling phase compared to the non-labeled period (11.88 ± 0.20 g L^–1^ vs. 11.32 ± 0.10 g L^–1^), *c*_X_ values proportionally increased to 6.20 ± 0.11 g L^–1^. However, microbial kinetics remained constant compared to the non-labeled state (Table 3). Labeling samples were taken after 3.8 RTs in 10 min intervals to document the expected isotopic stationarity. Sample processing followed the protocol given in the Supplementary Material sections “Sampling, Quenching, and Extraction for Quantification of ^13^C-Isotopic Labeling Dynamics” and “LC-MS-Based Quantification of Intracellular Pool Concentrations and ^13^C-Labeling Dynamics.”

Biomass-specific extracellular rates were furthermore estimated using a bioprocess model (see Supplementary Material section “Bioprocess Model Used for External Rate Estimation”). The rate of glucose consumption was estimated to be *r_GLC_* = 4,130 ± 150 μmol g_CDW_^–1^ h^–1^, the specific growth rate as μ = 0.40 ± 0.03 h^–1^, CO_2_ production and PCA uptake rates were determined as *r*_CO__2_ = 8,240 ± 760 μmol g_CDW_^–1^ h^–1^ and *r_PCA_* = 13 ± 1 μmol g_CDW_^–1^ h^–1^, respectively.

From these rates and the (corrected) MIDs, extracellular and intracellular fluxes were estimated with the ^13^C-MFA model at hand (see section “Metabolic Network Model for ^13^C-MFA”), following the procedure described in section “External Rate Estimation.” For the best fit obtained, measured and simulated data agreed very well (Supplementary Figures 3,4), as indicated by a SSR of approximately 31. Therewith the SSR was lower than the maximally acceptable SSR of 114 at 95% significance level. The estimated absolute fluxes including flux standard deviations are provided in the Supplementary Table 2.

**FIGURE 8:**
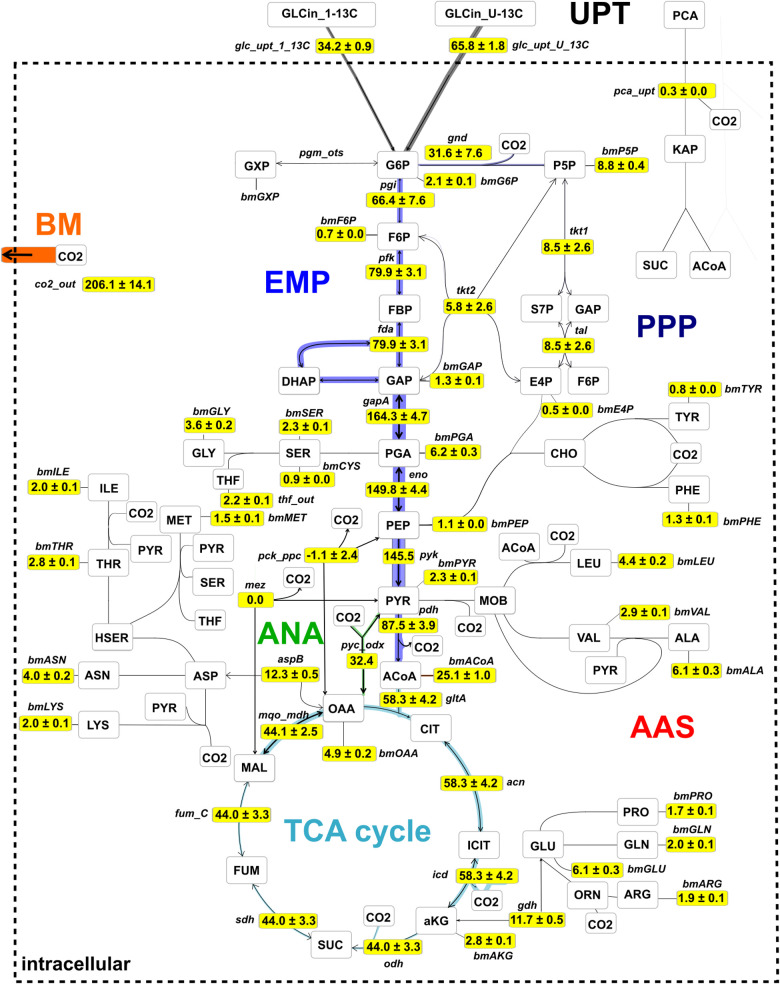
Metabolic flux map for *C. glutamicum* WT cultivated under continuous conditions at growth rate μ = 0.41 h^– 1^ using glucose as main carbon source. Fluxes were estimated using isotopic stationary ^13^C-MFA with a data set generated in a CLE with 33% (1-^13^C)- and 67% (U-^13^C)-D-glucose. Shown are net flux values, normalized to glucose uptake rate (*v*_GLC_ = 4,086 μmol g_CDW_^– 1^ h^– 1^), together with their standard deviations. The complete absolute and relative flux distributions are provided in the Supplementary Table 2. The flux map is drawn using the network editor and visualization tool Omix (Omix Visualization GmbH, Lennestadt, Germany, www.omix-visualization.com). UPT, uptake; PPP, pentose-phosphate pathway; ANA, anaplerotic section; BM, biomass; EMP, Embden–Meyerhof–Parnas; AAS, amino acid synthesis; TCA, tricarboxylic acid.

Figure 8 shows the central metabolic carbon fluxes, normalized to glucose consumption rate *v*_GLC_ = 4,086 μmol g_CDW_^–1^ h^–1^ for the specific growth rate μ = 0.41 ± 0.02 h^–1^. Two-thirds (66.4 ± 7.6%) of the incoming glucose flux were metabolized via the EMP pathway through G6P isomerase (reaction ***pgi***), whereas one-third (31.6 ± 7.6%) of the glucose was metabolized via the PPP through G6P dehydrogenase and phosphogluconate dehydrogenase (***gnd***) to regenerate nicotinamide adenine dinucleotide phosphate (NADPH) and accomplish P5P-precursor production for nucleotides and amino acids. A considerable portion of the input flux, namely 25.1 ± 1.2%, was utilized for acetyl-CoA precursors, e.g., the synthesis of fatty acids and to satisfy the demand for biomass. A relatively high proportion (58.3 ± 4.2%) of the incoming glucose was channeled into the TCA cycle via citrate synthase (***gltA***) to generate ATP via the respiratory chain and to satisfy the demand of amino acids of the ASP and GLU branches, whereas the contribution of the PCA flux to the TCA cycle via the β-keto-adipate pathway was negligibly small (*v*_PCA_ = 0.3 ± 0.0%). Best fit results suggest that the anaplerotic net flux mainly goes through ***pyc***, which channels 31.3% of the glucose uptake flux into the TCA cycle via oxaloacetate (OAA). In *C. glutamicum*, three reactions in central carbon metabolism, namely, isocitrate dehydrogenase (***icd***), ***gnd***, and malic enzyme (***mez***) contribute to regeneration of NADP^+^ to NADPH (Marx et al., 1996). According to the flux map (Figure 8), ***gnd*** (1,290 ± 309 μmol g_CD__W_^–1^ s^–1^) and ***icd*** (2,380 ± 169 μmol g_CDW_^–1^ s^–1^) contribute in a roughly 1:2 proportion to NADPH generation. Because the TCA cycle contributes 1 NADPH molecule per CIT molecule and the oxidative PPP contributes 2 NADPH molecules per G6P molecule, flux estimations suggest that NADPH demands were equally met by ***icd*** (48% of NADPH) and the lumped reactions of the oxidative PPP (52% of NADPH), recognizing that the contribution of ***mez*** is negligible. From the flux map in Figure 8 it follows that the total relative flux of NADPH synthesis is approximately 121.5% of the glucose consumption rate. Assuming the coefficient for NADPH required for growth to be 14,849 μmol g_CDW_^–1^ (Neidhardt et al., 1990), NADP^+^ has to be regenerated at a relative rate of 149.0%, leaving a gap of 27.5% molar flux relative to the glucose consumption rate. Notice that the net ***mez*** flux is statistically non-identifiable (Supplementary Table 2). A value of approximately 1,144 μmol g_CD__W_^–1^ s^–1^ suffices to close the gap, whereas any larger value results in excess NADPH production. Finally, the analysis showed that the total carbon flux into the biomass and CO_2_ was 65.6 and 34.4%, respectively, where 1 mol of glucose was converted into 2.06 mol CO_2_. Therewith, trends of relative fluxes are similar to those reported by previous studies at slightly higher specific growth rates (μ = 0.42, 0.46 h^–1^) and glucose uptake rates (*v*_GLC_ = 4,800, 5,100 μmol g_CDW_^–1^ h^–1^) conducted under batch conditions (Becker et al., 2011; Wang et al., 2018; see Supplementary Figure 5). Accordingly, biomass yields per glucose obtained in these studies are comparable with this study (*Y*_X,GLC_ = 0.49 and 0.50 g g_CDW_^–1^, respectively; see also Eq. 10 in Supplementary Material section “Bioprocess Model Used for External Rate Estimation”).

## Discussion

### Kinetic Studies

Figures 1, 2 together with Tables 1, 3 give evidence to the high repeatability of biological and technical replicates qualifying the data sets as sound and representative. The growth de-coupled maintenance coefficient, *m*_S_ is identified as (0.44 ± 0.04) mmol D-glucose g_CDW_^–1^ h^–1^ and the growth-coupled maintenance coefficient ${\text{Y}}_{\text{XS}}^{\text{real}}$ as (0.103 ± 0.01) g_CDW_ mmol^–1^ which indicates about 9 and 1% standard deviation, respectively. Surprisingly, the *m*_S_ value exceeds measurements of Koch-Koerfges et al. (2013) by factor 5.5 whereas ${\text{Y}}_{\text{XS}}^{\text{real}}$ of the current study is about 21% lower. Accordingly, results of the current study hint to higher cellular maintenance needs which consequently lead to reduced glucose-to-biomass conversion. As strains and media compositions are similar, no apparent reason is found for the discrepancy. However, determined growth kinetics which built the basis for maintenance calculations in this study agree well with kinetics determined by Cocaign-Bousquet et al. (1996) investigating *C. glutamicum* WT ATCC 17965 in chemostat tests.

### Metabolic and Transcriptional Regulation of Central Metabolism in Chemostat

The metabolic flux map of Figure 8 mirrors cellular efforts to provide sufficient metabolic precursors, reduction equivalents, and ATP at a growth rate of 0.4 h^–1^ that represents growth conditions typically observed under non-limited batch conditions (Blombach et al., 2013; Grünberger et al., 2013; Graf et al., 2018; Haas et al., 2019). Apparently, the central metabolism of *C. glutamicum* serves the pivotal need of precursor supply for biomass formation which is mirrored by the strong activity of glycolysis fueling the TCA cycle. Somewhat inferior are cellular needs for NADPH which is disclosed by only 1/3 of consumed glucose branching into the PPP thereby meeting half of the demands required for nucleotide precursors, biomass, and growth.

Pool sizes of upper glycolysis and PPP rise with the growth rate (G6P, F6P, FBP, DHAP) whereas intracellular concentrations of 2,3PG and PEP show opposite trends (see Figure 3). Notably, 2/3PG assigns the lumped pool of 2PG + 3PG of low total size which may be discarded from further discussion. Glycolytic fluxes are expected to increase with growth considering raising biomass specific glucose consumption rates of Table 1. Noack et al. (2017) found constant enzyme concentrations in central metabolism of *C. glutamicum* ATCC 13032 cultivated in the same CGXII minimal medium and under the same process conditions in chemostat mode (*D* = 0.05, 0.1, 0.2, and 0.35 h^–1^) as employed in this study. Our findings agree with the previous study because only gene *pgi* showed minor reduction of transcript levels of genes encoding enzymes in glycolysis and in PPP. Consequently, increasing glycolytic fluxes should be enabled by elevated intracellular pool concentrations fairly assuming Michaelis–Menten type kinetics for said enzymes. Because extracellular glucose concentrations were limiting under chemostat operation, the majority of glucose consumption is expected to happen via the PTS. PEP is dephosphorylated stoichiometrically to pyruvate (not measured) activating glucose to G6P. Accordingly, increasing PEP consumption coincides with raising glucose consumption rates and growth. Observing falling PEP pools anticipate limited PEP regeneration with increasing growth rate. In general, glycolytic metabolite dynamics agree with independent studies of Noack et al. (2017) hinting to metabolically controlled glycolytic fluxes in glucose-limited chemostats.

In the TCA cycle, metabolite dynamics are less pronounced than in glycolysis and PPP. The strongest increase found in the lumped CIT/ICIT pool might be misleading as the low pool sizes reflect the particular instability of these metabolites using the leakage-reduced fast centrifugation treatment and hot-water extraction protocol for separation and metabolite extraction. However, elevating pools with growth may also mirror increased citrate synthase catalyzed flux (indicated by amplified *gltA* expression; van Ooyen et al., 2011) which is not passed on to aKG because of missing *icd* amplification (Figure 4). Rising MAL and FUM levels may mirror rising fluxes catalyzed via Michaelis–Menten type kinetics of fumarate hydratase, malic enzyme and malate:quinone oxidoreductase whose corresponding genes (*fum*, *malE*, *mqo*; Han et al., 2008b) did not show any changes of expression levels. Interesting enough the expression of *pck*, encoding phosphoenolpyruvate carboxykinase (Riedel et al., 2001), reduces with growth which agrees with the hypothesis of limited PEP regeneration from OAA.

In general, the identification of only a few DEGs in TCA cycle agrees with findings of Noack et al. (2017) analyzing the enzyme content of *C. glutamicum*. Similar to glycolysis, growth dependent TCA cycle activity appears to be mostly metabolically controlled under chemostat conditions. Rising *gltA* expression, however, may thus be qualified as one example of transcriptional control. In case of the other DEGs, a coherent regulatory scheme is missing so far. Furthermore, putative regulator genes such as *ramA* and *sugR* are unlikely to exert additional control as they show only moderate dynamics with increasing growth rate (Figure 6).

Most of the amino acid pools show rising sizes with increasing growth (Supplementary Figure 1) which reflects increasing needs of said precursors for protein synthesis using the assumption of Michaelis–Menten type enzyme kinetics. Frankly speaking, there are no obvious reasons why MET and LEU pools fall. However, the observation of growth rate independent intracellular pool sizes of ASP, ASN, and GLU agrees with findings of Graf et al. (2018). The authors outlined that those amino acids were consumed non-proportionally with the strain’s amino acid biomass composition under non-limited glucose and amino acid conditions (batch cultivations). They concluded that most likely, these compounds serve as central amino donors for multiple acceptors explaining why their uptake outnumbers stoichiometric biomass needs by far.

### Comparability of Chemostat and Batch Observations

Haas et al. (2019) identified the so-called “growth modulon” of *C. glutamicum* analyzing the growth in the same medium as in this study, but after preceding installation of carbon, nitrogen, and phosphate limitation. The intersection analysis of DEGs changing with accelerating growth rates after previous stress exposure yielded a set of 447 genes belonging to the growth modulon. Noteworthy, the batch study of Haas et al. (2019) differs from chemostat tests by installing saturating nutrient levels including glucose whereas glucose limitation and low extracellular glucose concentrations occur in the continuous runs. Interesting enough, Haas et al. (2019) analyzed transcriptional patterns at the growth rates 0.2, 0.3, and 0.4 h^–1^ which are identical to the chemostat growth rates of the current study. This allows the direct comparison of transcriptional patterns associated to equal growth but under different glucose levels.

A primary observation is that changes of gene expression levels are more pronounced under batch conditions (Haas et al., 2019) than in chemostat mode. On average, log fold changes (LFCs) of batch DEGs are twice as large as those of the continuous culture. Apparently, non-transcriptional control of cellular growth is more prominent in chemostat than in glucose-rich batch conditions. Interestingly, most of the genes coding for TCA cycle reactions are not members of the “growth modulon” which agrees well with the proteome measurements of Noack et al. (2017) and with the chemostat transcript results of this study. In contrast, acetate kinase (encoded by *ackA*) and phosphate acetyltransferase (encoded by *pta*) showed amplifying trends (Figure 4) and are part of the growth modulon. Consequently, both conditions, chemostat, and batch, assigned those genes identical functions, either as part of the non- or as member of the growth correlated fractions.

Contrary, *aceA* (isocitrate lyase), *pqo* (pyruvate:quinone oxidoreductase), and *sucCD* (succinate CoA ligase) revealed opposite expression trends in chemostat runs compared to the expectations as members of the growth modulon derived from batch observations. Even more striking, the entire glycolysis was assigned to the growth modulon anticipating increasing transcripts with rising growth. However, transcriptome (Figure 5) and proteome (Noack et al., 2017) studies of chemostats did not depict such dynamics. Apparently, findings of batch studies may not be transferred one-by-one to chemostat conditions, *vice versa*.

For explanation, Figure 6 should be discussed: considering that extracellular glucose levels were below the detection limit in chemostats, cells may require stronger amplification of *iolT1* (coding for glucose specific EII permease; Ikeda et al., 2011; Lindner et al., 2011) than under high-glucose batch conditions. Notably, early studies of Cocaign-Bousquet et al. (1996) followed by glucose uptake engineering works of Xu et al. (2019) outlined that PTS-based glucose imports dominate glucose uptake by 70–85% in *C. glutamicum*. Accordingly, non-PTS glucose import may account for roughly 15–30%. Nevertheless, the finding of increasing *iolT1* levels with chemostat growth supports the hypothesis that non-PTS glucose uptake may have gained importance. *iolT1* is under control of *iolR* (Klaffl et al., 2013), *iolT2* is supposed to be regulated by cg3388 (Brinkrolf et al., 2006). By trend, the second correlation is observed whereas the first is not. Maybe, *iolT1* expression is co-controlled by another regulator not discovered yet. Glucose entering the cell via IolT1 requires activation by glucokinase either encoded by *glk* (Park et al., 2000) or by *ppgk* (Lindner et al., 2010). The ATP dependent glucokinase reveals slightly rising gene expression with growth whereas expression levels of the PolyP/ATP-dependent glucokinase reduce in continuous culture. Interestingly, *glk* levels in chemostat are mostly below those of glucose-rich batch conditions anticipating that permease-driven import of glucose is higher when extracellular levels are elevated too. Most remarkably, *ramA*, encoding one of the key regulators of the growth modulon (Haas et al., 2019) revealed only low expression in each chemostat environment whereas higher, amplifying trends were found in batch tests. As RamA controls expression of glycolytic genes (Auchter et al., 2011; Shah et al., 2018; Haas et al., 2019) their missing amplification in chemostats may be explained as the missing activation of *ramA*. Most likely, extracellular glucose levels somehow stimulate *ramA* activation but this remains to be shown.

## Conclusion

Investigating *C. glutamicum* WT under nutrient limiting conditions in chemostat, we found that pool sizes of glycolysis predominately rise with growth. Although not measured explicitly, rising intracellular fluxes are likely to follow the experimentally observed increasing, growth coupled glucose uptake rates. In particular, the scenario pinpoints to a predominant metabolic control of glycolysis because indications for transcriptional control could not be detected in chemostat. By analogy, growth coupled metabolic control of PPP and TCA cycle is anticipated. Again, concerted trends in transcriptional patterns could not be found. The picture is complemented by ^13^C-studies underlining the central function of central metabolism as supplier of precursors, redox, and energy equivalents.

As such, the study consolidates previous findings outlining a particularity: glycolysis apparently is transcriptionally controlled under glucose-rich batch condition whereas metabolic control dominates in glucose limited chemostats. The important player may be RamA, a key transcriptional regulator of glycolysis. Obviously, it is a highly attractive goal of future studies to unravel the metabolic effector interacting with RamA, one of the remaining puzzle pieces to complete the growth regulation picture of *C. glutamicum*.

## Data Availability Statement

The datasets presented in this study can be found in online repositories. The names of the repository/repositories and accession number(s) can be found below: https://www.ebi.ac.uk/arrayexpress/, E-MTAB-9371.

## Author Contributions

MG designed the study, carried out the bioreactor experiments, analyzed the metabolic datasets, and drafted and corrected the manuscript. TH designed the study, analyzed the transcriptomic data sets and, drafted and corrected the manuscript. AT and AF conceptualized and performed metabolic analysis and corrected the manuscript. TB and JK measured the transcript samples and corrected the manuscript. MC and KN designed the flux study, performed the MFA, and drafted and corrected the manuscript together with WW. RT conceptualized the whole study and drafted and corrected the manuscript. All authors read and approved the final manuscript.

## Conflict of Interest

The authors declare that the research was conducted in the absence of any commercial or financial relationships that could be construed as a potential conflict of interest.

## Abbreviations

2/3PG: 2-/3-phosphoglyceric acid
aKG: alpha ketoglutarate
ASN: L-asparagin
ASP: L-aspartate
ATP: adenosine triphosphate
CDW: cell dry weight
CIT: citrate
CLE: carbon labeling experiment
DEG: differentially expressed gene
DHAP: dihydroxyacetone phosphate
EMP: Emden-Meyerhof-Parnas
F6P: fructose 6-phosphate
FBP: fructose 1,6-bisphosphate
FFC: flux fold change
FUM: fumarate
GAP: glyceraldehyde 3-phosphate
GLC: glucose
GLU: L-glutamate
ICIT: isocitrate
LEU: L-leucine
LFC: log fold change
MAL: L-malate
MET: L-methionine
MFA: metabolic flux analysis
MID: mass isotopomer distribution
NADPH: nicotinamide adenine dinucleotide phosphate
OAA: oxaloacetate
OD_600_: optical density
OED: optimal experimental design
P5P: pentose-5-phosphate
PEP: phosphoenolpyruvate
PCA: protocatechuic acid
PPP: pentose phosphate pathway
PRO: L-proline
RT: residence time
RNA: ribonucleic acid
S7P: sedoheptulose 7-phosphate
SUC: succinate
SSR: sum of squared residuals
TCA: tricarboxylic acid
TIC: total inorganic carbon
TOC: total organic carbon
WT: wild-type.
