# Supplementary material for: Revisiting the Growth Modulon of Corynebacterium glutamicum Under Glucose Limited Chemostat Conditions
Source: Front Bioeng Biotechnol. 2020 Oct 15;8:584614. doi: 10.3389/fbioe.2020.584614 (PMC7594717; doi:10.3389/fbioe.2020.584614)
Supplement: Supplementary file 1 [file Table_1.docx]

Supplementary Material

Revisiting the Growth Modulon of *Corynebacterium glutamicum* under Glucose Limited Chemostat Conditions

Michaela Graf^1a^, Thorsten Haas^1a^, Attila Teleki^1^, André Feith^1^, Martin Cerff^2^, Wolfgang Wiechert^2^, Katharina Nöh^2^, Tobias Busche^3,4^, Jörn Kalinowski^3^, Ralf Takors^1*^

^1^Institute of Biochemical Engineering, University of Stuttgart, Allmandring 31, 70565 Stuttgart, Germany

^2^Institute of Bio- and Geosciences, IBG-1: Biotechnology, Forschungszentrum Jülich GmbH, Wilhelm-Johnen-Str., 52428 Jülich, Germany

^3^Center for Biotechnology (CeBiTec), Bielefeld University, Bielefeld, Germany

^4^Institute for Biology-Microbiology, Freie Universität Berlin, Berlin, Germany

*** Correspondence:**

Prof. Dr.-Ing. Ralf Takors

[takors@ibvt.uni-stuttgart.de](mailto:takors@ibvt.uni-stuttgart.de)

**^a^Both authors contributed equally to this work.**

# Material and methods for metabolome analysis

## Generation of fully ^13^C-labeled *C. glutamcium* extracts for IDMS

Pre-cultures from glass reaction tubes (main text section 2.1) were used to inoculate individual shaking flasks filled with 50 mL sterile modified CGXII medium (Buchholz et al., 2014) supplemented with 40 g L^-1^ [U-^13^C]- D-glucose as sole carbon source. In the late exponential growth phase after 12.5 h, stationary labeled cultures from three shaking flasks were pooled yielding a biomass concentration of 12 g_CDW_ L^-1^. The labeled biomass was harvested by centrifugation (5 min, 3000 g, 4°C; 5430 R, Eppendorf, Hamburg, Germany), washed with 9 g L^-1^ NaCl solution, and was centrifuged again. To extract labeled intracellular metabolites the adapted hot-water-extraction (HWE) procedure (section 1.3.1) was employed. Boiling (100 °C) MS-grade water (Carl Roth, Germany) was added to biomass pellets of 50 g_CDW_ L^-1^, incubated at 100 °C for 2 min in a water bath (Lauda RK20, Germany), and resuspended by short-time mixing. Incubation and mixing was repeated three times and the resulting suspension was chilled on ice water. Finally, labeled extracts were separated from cell debris by centrifugation (10 min, 20,000 x g; 5430 R, Eppendorf, Hamburg, Germany). Thereof aliquots were prepared and stored at -70°C until measurement (section 1.3.3).

## Analysis of exometabolome

Biomass formation in batch cultivation phase of continuous processes was followed by measuring the optical density (OD_600_, DR 3900, Dr. Lange, Berlin, Germany). In the continuous phase, biosuspension was withdrawn in 1.5 h intervals for three times after reaching a metabolic steady state at the respectively installed dilution rate ( main text section 2.2.2) to determine OD_600_, cell dry weight (CDW), and total inorganic carbon (TIC) species and to analyze glucose concentrations in cell-free filtrates (by-product formation was not observed in preliminary continuous experiments). For CDW samples, 3 mL (chemostat processes at *µ* = 0.2, 0.3, 0.4 h^-1^) or 1.5 mL (^13^C-labeling experiment) biosuspension was aliquoted into glass reactions tubes in technical quadruplicates, washed twice in deionized water and centrifuged between each washing step for 10 min at 3000 x g and 4 °C (5430 R, Eppendorf, Hamburg, Germany). The washed biomass was dried for at least 48 h in a convection oven (Heraeus, Hanau, Germany) at 105 °C. After cooling in a desiccator, tubes were weighed on a laboratory scale (AE 200, Mettler Toledo, Gießen, Germany). Cell-free samples were obtained by filtering biosuspension through 0.2 µm pore size syringe filters (Rotilabo®, Carl Roth, Karlsruhe, Germany) and stored at -20 °C until measurement. Glucose concentration was determined with an enzymatic assay following the manufacturer’s instruction (R-biopharma, Darmstadt, Germany). TIC amounts in biosuspension samples were determined as described by Buchholz et al. (2014) to correct for underestimated CO_2_-values measured in off-gas analysis.

## Analysis of endometabolome

### Sampling, quenching, and extraction for absolute quantification of intracellular pool concentrations

To quantify intracellular metabolite pools of *C. glutamicum*’s central carbon metabolism at different growth rates (*µ* = 0.2, 0.3, 0.4 h^-1^), fast centrifugation treatment (FCT) in combination with hot-water-extraction (HWE) was performed according to a leakage-reduced and sequential protocol of Teleki et al. (2015 and 2017). For fast centrifugation, 2 mL biosuspension was centrifuged at 20817 ×g and -2 °C for 30 s (5430 R, Eppendorf, Hamburg, Germany), the supernatant was discarded and the cell pellet was immediately frozen in liquid nitrogen. For hot-water-extraction (HWE), the protocol was slightly adapted. 30 μmol L^-1^ L-norvaline solution was added to the frozen cell pellets to obtain a biomass concentration of 50 g L^-1^. Subsequently, the suspension was incubated in a water bath at 100 °C for 1 min, thoroughly mixed, and again incubated for 5 min. Afterwards, samples were cooled on ice and centrifuged at 20817 ×g and 4 °C for 10 min. The metabolite-containing supernatant was carefully removed and aliquots were stored at -70 °C until measurement.

###
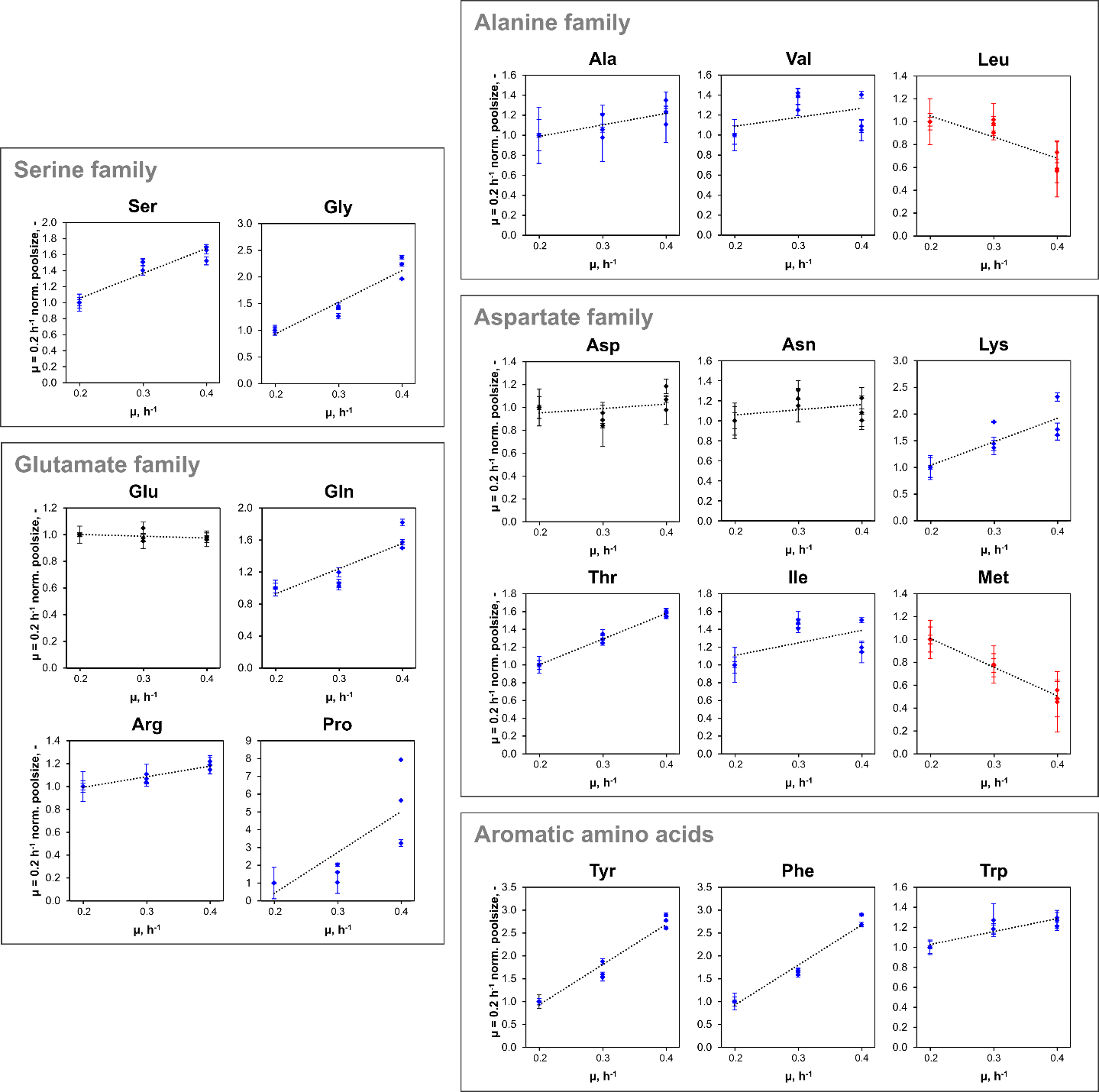
Intracellular pool concentrations of amino acids

Supplementary Figure 1: Intracellular amino acid pools of *C. glutamicum* WT as response to different growth rates (*µ* = 0.2 h^-1^, 0.3 h^-1^, 0.4 h^-1^) installed in three independent chemostat processes. Depicted intracellular metabolite concentrations were normalized to respective values determined at μ = 0.2 h^-1^; values represent the mean of three technical replicates ± standard deviation. Color code: red = decreasing concentration; blue: increasing concentration.

###

### Sampling, quenching and extraction for quantification of ^13^C-isotopic labeling dynamics

To follow the progress of ^13^C-labeled carbon through *C. glutamicum*’s carbon metabolism, samples were generated according to an adapted and sequential protocol of Koning and Dam (1992) employing rapid cold-methanol quenching (CMQ) and methanol-chloroform extraction (CME). 2 mL biosuspension was directly mixed after withdrawal from the bioreactor with 3 mL of 60 % (v/v) pre-cooled (-70 °C) methanol solution. The mixture was centrifuged at 4000 × g and -2 °C for 10 min (5430 R, Eppendorf, Hamburg, Germany) and the residual biomass pellet was frozen in liquid nitrogen. Until further treatment, all samples were stored at -70 °C. Frozen cell pellets were fully resuspended in 1 mL 50% (v/v) pre-cooled (-20 °C) aqueous methanol solution. Afterwards, 1 mL pre-cooled (-20 °C) chloroform was added, and the suspension was mixed and incubated for 2 h at -20 °C in an overhead-shaker. After centrifugation of samples at 3824 ×g and -2 °C for 10 min, the upper aqueous methanol phase containing polar intracellular ^13^C-labeled metabolites was carefully removed and aliquots were stored at -70 °C until measurement.

### LC-MS-based quantification of intracellular pool concentrations and ^13^C-labeling dynamics

LC-MS analyses of intracellular metabolite extracts (sections 1.3.1 and 1.3.2) were performed as previously described in a cross-platform ^13^C-tracer quantification study (Feith and Teleki et al., 2019). Both methods were based on a bicratic zwitterionic hydrophilic interaction chromatography (ZIC-pHILIC) under alkaline mobile phase conditions (Teleki et al. 2015). Targeted metabolome analyses were conducted on an Agilent 1200 HPLC system coupled with an Agilent 6410B triple quadrupole mass spectrometer (MS/MS-QQQ). Non-labeled intracellular pools were absolutely quantified by isotope dilution mass spectrometry (IDMS) and a constant addition of [U-^13^C]-labeled *C. glutamicum* extracts (section 1.1) in multiple reaction monitoring (MRM) mode with a mass resolution of 0.1 u. Non-targeted metabolome analyses were conducted on an Agilent 1260 HPLC system coupled with an Agilent 6540 quadrupole time-of-flight high-resolution mass spectrometer (QTOF-HRMS). Isotopic distributions of ^13^C-labeled and non-labeled intracellular pools of analogues samples were quantified in MS mode with a scan range of m/z 25-1700.

# ^13^C-MFA

## Model setup

The ^13^C-MFA model used for flux estimation was taken from (Kappelmann et al., 2016) and modified as follows (supplementary FluxML file ‘CoryneWT_STAT_MFA_FIT.fml’):

- an unidirectional PCA uptake reaction was added via keto-adipate into the TCA cycle
- the three pentose phosphate pools X5P, Ru5P, R5P were lumped into one pool P5P; the reactions ***rpi***, ***rpe*** were removed and the biomass efflux was remodeled accordingly
- nucleotides (IMP, UMP) were removed
- the pool G1P was added to the glycogen pathway
- the lumped pool CIT/ICIT was replaced by two separate pools CIT, ICIT connected bidirectionally
- the pools SUCCOA, SUC were lumped
- the intermediate pool DAP was eliminated from the lysine synthesis pathway
- the ***gdh*** reaction was modeled as reversible reaction
- ARG synthesis was added and ORN introduced
- TRP and HIS were removed
- an additional pool CHO was considered for the synthesis of aromatic amino acids PHE, TYR
- ***pyc_odx*** and ***mez*** were assumed to operate unidirectionally

The ^13^C-MFA model used for optimal tracer design differs from the flux estimation model in minor points (supplementary FluxML file ‘CoryneWT_STAT_MFA_OED.fml’):

- no PCA uptake
- no G1P pool
- contains ACN
- three pentose phosphate pools (X5P, Ru5P , R5P), bidirectionally connected
- no ARG and ORN pools
- unidirectional ***icd***, ***acn*** and ***gdh*** reactions
- non-symmetric ***lys_CA*** reaction

## Optimal experimental tracer design

To design optimally informative isotope labeling experiments, optimal experimental design (OED) studies were performed considering six D-glucose (GLC) tracer species:

- [U-^12^C]- GLC
- [U-^13^C]- GLC
- [1-^13^C]- GLC
- [1,2-^13^C]- GLC
- [6-^13^C]- GLC
- [5,6-^13^C]- GLC

In total, 8,000 compositions of these species were evaluated, rated according to the covariance based A-criterion as flux information measure (Möllney et al., 1999). Results were averaged over flux distributions extrapolated from previous flux analyses (Supplementary Table 1). Selected mixture triangles are shown in supplementary figure 2. The assumptions made concerning the envisioned measurements are detailed below.

Supplementary Table 1: Reference flux maps used for tracer design.

| Flux name | | A  mmol g_CDW_**^-1^** h**^-1^** | B  mmol g_CDW_**^-1^** h**^-1^** |
| --- | --- | --- | --- |
| ***glc_upt*** | net | 1.80 | 1.68 |
| ***gnd*** | net | 1.22 | 1.46 |
| ***mqo_mdh*** | net | 2.06 | 0.64 |
| ***mu_mes*** | net | 0.20 | 0.20 |
| ***pyk*** | net | 1.82 | 0.00 |
| ***aspB*** | xch | 0.99 | 0.99 |
| ***eno*** | xch | 32.97 | 3.79 |
| ***fda*** | xch | 0.10 | 0.10 |
| ***fumC1*** | xch | 0.60 | >1,000 |
| ***gapA*** | xch | >1,000 | >1,000 |
| ***mez*** | xch | 0.00 | 0.06 |
| ***mqo_mdh*** | xch | >1,000 | 0.01 |
| ***pck_ppc*** | xch | 0.00 | 0.59 |
| ***pgi*** | xch | >1,000 | 16.00 |
| ***pyc_odx*** | xch | 0.00 | 0.00 |
| ***rpe*** | xch | 5.54 | 4.11 |
| ***rpi*** | xch | 9.25 | >1,000 |
| ***sdh2*** | xch | >1,000 | >1,000 |
| ***tal*** | xch | 0.17 | 0.03 |
| ***tkt1*** | xch | 14.86 | 4.32 |
| ***tkt2*** | xch | 0.15 | 0.00 |
| ***tpi*** | xch | 2.46 | 1.43 |


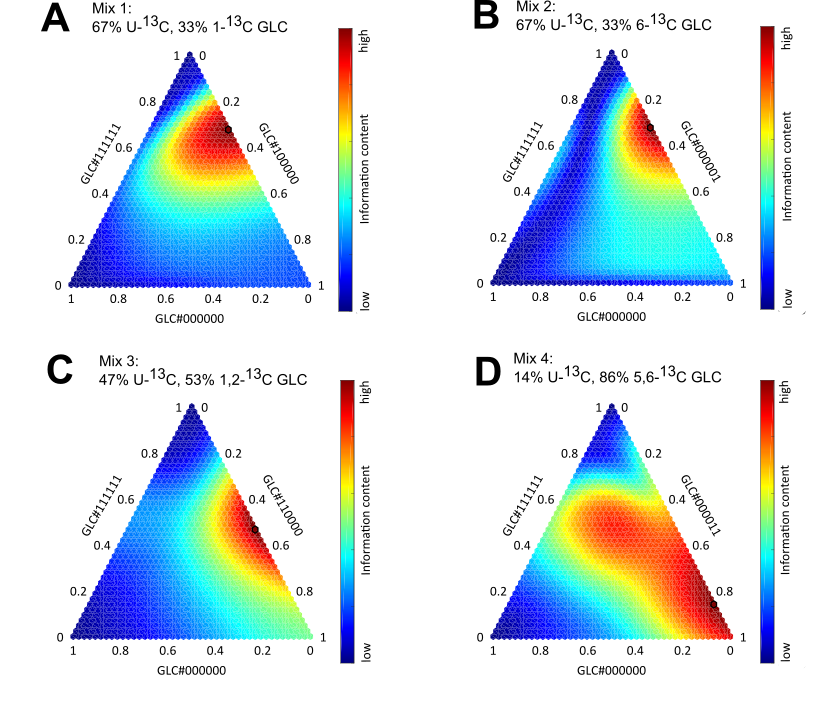


Supplementary Figure 2: Substrate mixture triangles. Exemplary results of the experimental design study showing the outcome for triple mixtures of commonly used isotopically labeled glucose species. The colors indicate the information criterion (averaged expected flux variances). Red and blue areas depict mixtures with high and low flux information, respectively. Overall, the composition in (A), 0 % naturally ^12^C-, 33 % 1-^13^C- and 67 % U-^13^C-labeled D-glucose performed best in terms of expected flux information and under consideration of the substrate costs. Costs per gram glucose of the depicted information optimal mixtures are 157 € (Mix 1), 911 € (Mix 2), 549 € (Mix 3) and 2039 € (Mix 4) (costs according to Sigma Aldrich, inquired 2016 for a 99 atom % purity).

Generally, tracer design relies on the specification of metabolite fragments that are expected to be measurable in the ILE, together with their expected errors.

For the labeling measurements we made the following assumptions according to prior work (Feith et al., 2019; Teleki et al., 2015):

- MS/MS fragment information for these metabolites:
- ACN, AKG, ALA, ASP, ASN, FUM, HSER, GLN, GLU, GLY, ILE, LEU, MET, PHE, PRO, SER, SUC, THR, TYR, VAL
- MS fragment information for the metabolites
- DHAP, FBP, F6P, G6P, MAL, PEP, PGA, S7P, R5P, RU5P, X5P

The associated expected measurement errors were determined by error models. These error models are linear functions of the signal intensity, as previously determined (Nöh et al., 2018):

- MS: 0.007797+0.009040*meas [-]
- MS/MS: 0.001696+0.016496*meas [-]

For the transport rates, as for comparable experiments, we assume these rates to be measurable: *µ*, *r_GLC_*_,_ *r_CO2_* with expected relative errors of 3.4%, 5.0%, and 3.0 %, respectively.

The complete measurement specification is provided in ‘CoryneWT_STAT_MFA_OED’.

## Flux estimation


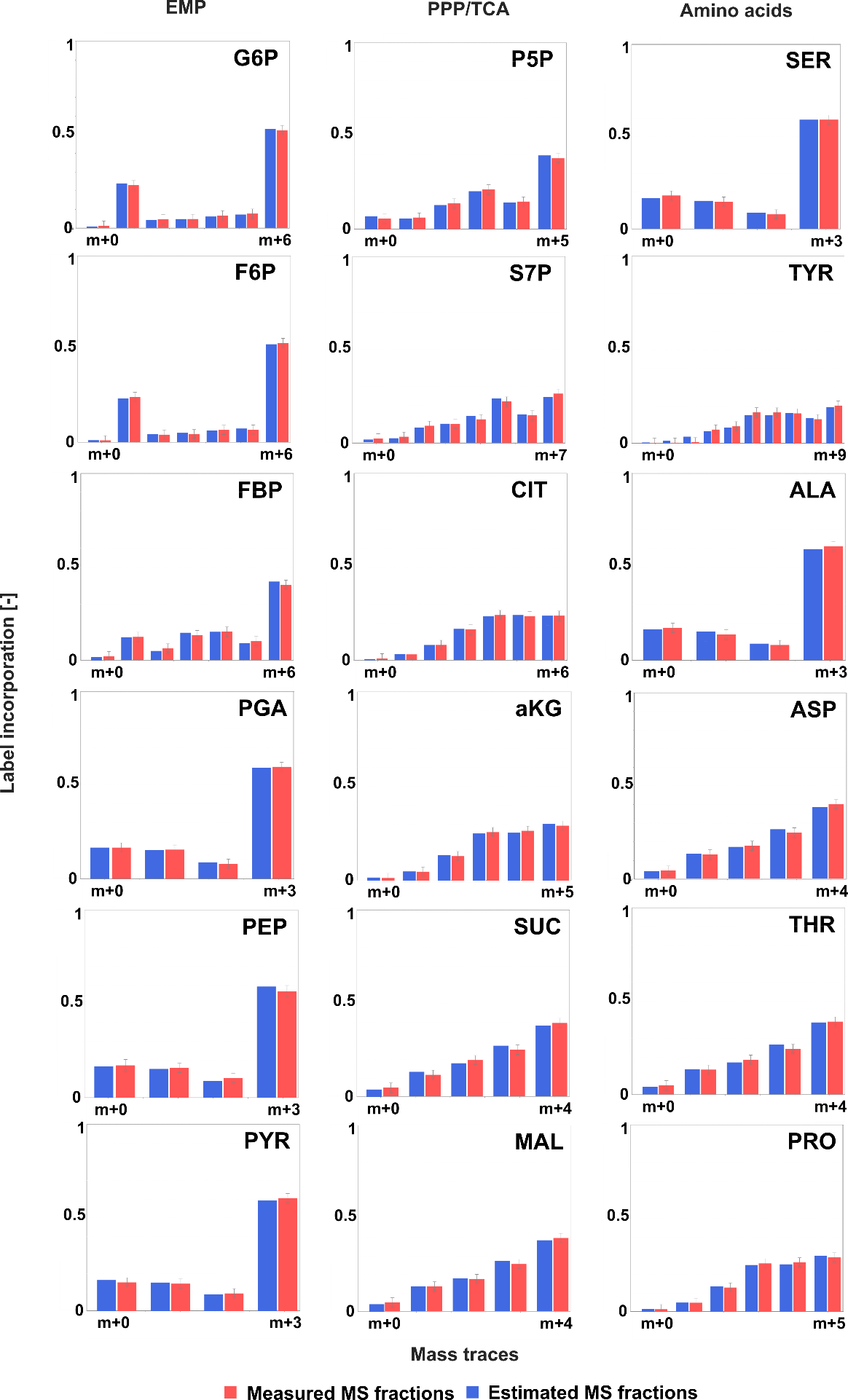


Supplementary Figure 3: Measured versus model-predicted labeling patterns. Measured (red) and simulated (blue) mass isotopomer distributions (MID) for selected metabolites of the EMP, PPP and TCA pathways as well as free amino acids. For the measurements, error bars indicate standard deviations, employed in this study.


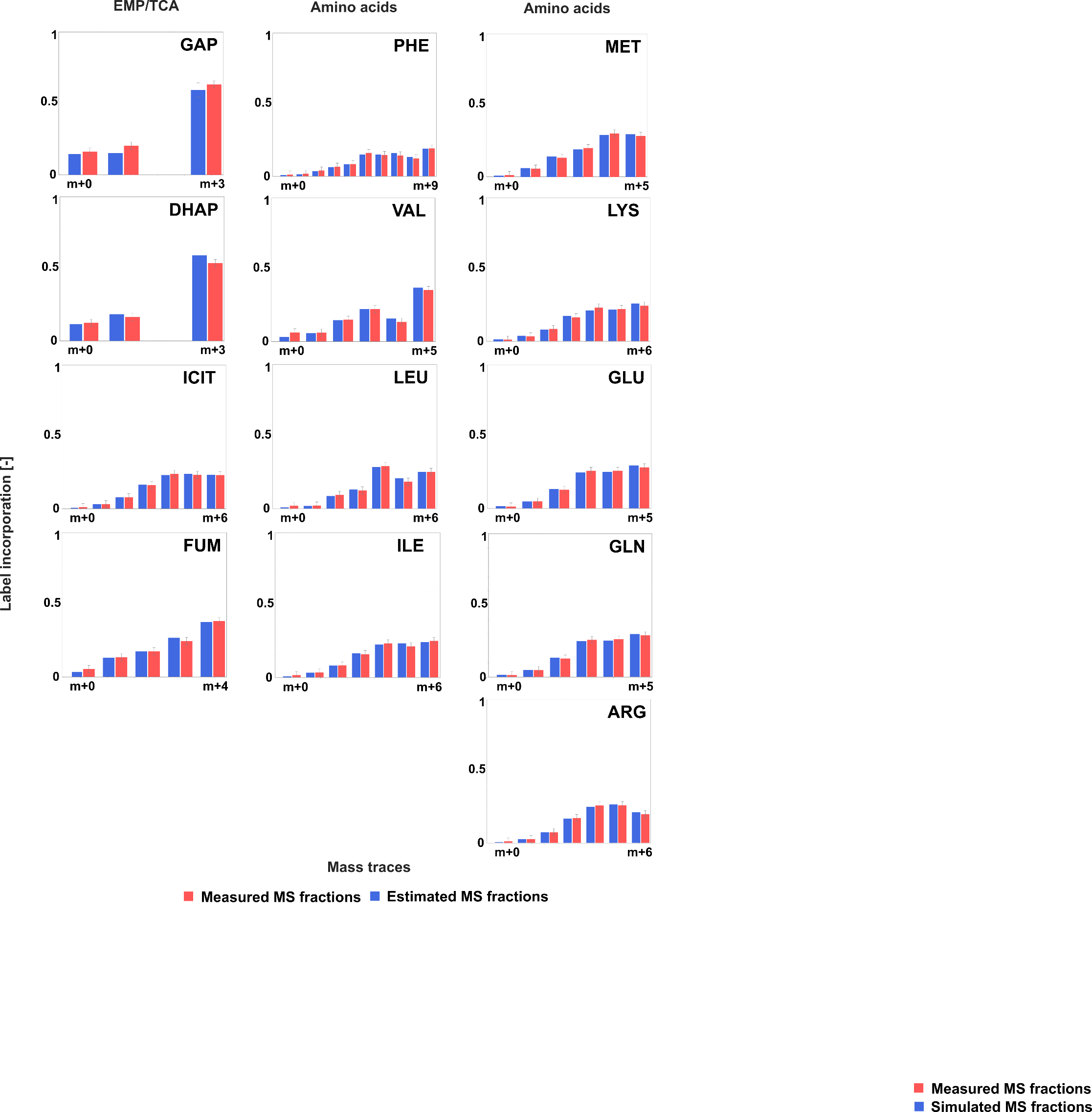


Supplementary Figure 4: Measured versus model-predicted labeling patterns. Measured (red) and simulated (blue) mass isotopomer distributions (MID) for selected metabolites of the EMP, PPP and TCA pathways as well as free amino acids. M+2 measurements of GAP and DHAP were discarded as unreliable and omitted for fitting. For the measurements, error bars indicate standard deviations, employed in this study.

Supplementary Table 2: Reaction network with carbon mappings and reaction (bi)directionalities, absolute and relative fluxes, in net and exchange flux coordinates, lower and upper bounds (LB, UB); “nd”: not determinable; n=net flux, x=exchange flux.

| Reaction name | Reaction |  | Abs. fluxes [µmol/g/h] | |  | STD [µmol/g/h] | Rel. fluxes [% uptGLC] | STD [% uptGLC] | LB/UB [µmol/g/h] | |
| --- | --- | --- | --- | --- | --- | --- | --- | --- | --- | --- |
| *acn* | CIT(abcdef) ↔ ICIT(afcbed) | n. | 2380.2 |  |  | 168.7 | 58.3 | 4.2 |  | |
|  |  | x. | 11974.2 |  |  | - | 292.4 | - | [0, 5e6] |  |
| *argB* | GLU(abcde) + CO2(f) → ORN_CIL(badcef) | n. | 77.7 |  |  | 3.0 | 2.2 | 0.1 |  |  |
| *argGH* | ORN_CIL(abcdef) → ARG(abcdef) | n. | 77.7 |  |  | 3.0 | 1.9 | 0.1 |  | |
| *aro* | PEP(abc) + PEP(def) + E4P(ghij) → CHO(dhjaebigfc) | n. | 87.9 |  |  | 3.4 | 1.9 | 0.1 |  | |
| *aspB* | OAA(abcd) ↔ ASP(abcd) | n. | 502.9 |  |  | 19.4 | 12.3 | 0.5 |  | |
|  |  | x. | 5689.8 |  |  | - | 139.3 | - | [0, 1e5] | |
| *eno* | PGA(abc) ↔ PEP(abc) | n. | 6120.2 |  |  | 150.5 | 149.8 | 4.4 |  | |
|  |  | x. | 212285.5 |  |  | - | 5195.6 | - | [0, 5e6] | |
| *fda* | FBP(abcdef) ↔ GAP(dac) + DHAP(ebf) | n. | 3264.2 |  |  | 115.3 | 79.9 | 3.1 |  | |
|  |  | x. | 7061.1 |  |  | 2292.1 | 172.8 | 56.2 | [0, 5e5] | |
| *fumC_1* | FUM(abcd) ↔ MAL(badc) | n. | 900.6 |  |  | 91.1 | 22.0 | 2.3 |  | |
|  |  | x. | 68227.7 |  |  | - | 1669.8 | - | [0, 5e5] | |
| *fumC_2* | FUM(abcd) ↔ MAL(abcd) | n. | 900.6 |  |  | 91.1 | 22.0 | 2.3 |  | |
|  |  | x. | 68227.7 |  |  | - | 1669.8 | - | [0, 5e5] | |
| *gapA* | GAP(abc) ↔ PGA(bca) | n. | 6711.4 |  |  | 159.8 | 164.3 | 4.7 |  | |
|  |  | x. | 81465.6 |  |  | - | 1993.8 | - | [0, 5e6] | |
| *gdh* | aKG(abcde) ↔ GLU(abcde) | n. | 478.7 |  |  | 18.4 | 11.7 | 0.5 |  | |
|  |  | x. | 2080532.5 |  |  | - | 50919.5 | - | [0, 5e6] |  |
| *glc_upt_1_13C* | GLCin_1_13C(abcdef) → GLC(abcdef) | n. | 1395.4 |  |  | 31.2 | 34.2 | 0.9 |  | |
| *glc_upt_U_13* | GLCin_U_13C(abcdef) → GLC(abcdef) | n. | 2690.5 |  |  | 57.4 | 65.8 | 1.8 |  | |
| *glc_upt* | GLC(abcdef) → G6P(abcdef) | n. | 4085.9 |  |  | 65.3 | 100.0 | 2.3 | [3000, 4730] | |
| *glnA* | GLU(abcde) → GLN(abcde) | n. | 80.5 |  |  | 3.1 | 2.0 | 0.1 |  | |
| *gltA* | ACoA(ab) + OAA(cdef) → CIT(acbefd) | n. | 2380.2 |  |  | 168.7 | 58.3 | 4.2 |  | |
| *glyA* | SER(abc) → GLY(bc) + THF(a) | n. | 148.3 |  |  | 5.7 | 3.6 | 0.2 |  | |
| *gnd* | G6P(abcdef) → P5P(eadbc) + CO2(f) | n. | 1289.7 |  |  | 309.0 | 31.6 | 7.6 | [400, 2400] | |
| *icd* | ICIT(abcdef) ↔ aKG(badcf) + CO2(e) | n. | 2380.2 |  |  | 168.7 | 58.3 | 4.2 |  | |
|  |  | x. | 732.0 |  |  | - | 17.9 | - | [0, 5e6] | |
| *ilvAE* | THR(abcd) + PYR(efg) → ILE(aebfcd) + CO2(g) | n. | 83.0 |  |  | 3.2 | 2.0 | 0.1 |  | |
| *ilvB* | PYR(abc) + PYR(def) → MOB(daebc) + CO2(f) | n. | 297.5 |  |  | 11.5 | 7.3 | 0.3 |  | |
| *ilvEa* | VAL(abcde) + PYR(fgh) → ALA(fgh) + MOB(abcde) | n. | 249.0 |  |  | 9.6 | 6.1 | 0.3 |  | |
| *ilvEv* | MOB(abcde) → VAL(abcde) | n. | 365.7 |  |  | 14.1 | 9.0 | 0.4 |  | |
| *leuAP* | MOB(abcde) + ACoA(fg) → LEU(badcfg) + CO2(e) | n. | 180.8 |  |  | 7.0 | 4.4 | 0.2 |  | |
| *ltsA* | ASP(abcd) → ASN(abcd) | n. | 163.9 |  |  | 6.3 | 4.0 | 0.2 |  | |
| *lysCA_1* | ASP(abcd) + PYR(efg) → LYS(ceafbd) + CO2(g) | n. | 41.5 |  |  | 1.6 | 1.0 | 0.0 |  | |
| *lysCA_2* | ASP(abcd) + PYR(efg) → LYS(caebfg) + CO2(d) | n. | 41.5 |  |  | 1.6 | 1.0 | 0.0 |  | |
| *lysC_hom* | ASP(abcd) → HSER(acbd) | n. | 256.0 |  |  | 9.9 | 6.3 | 0.3 |  | |
| *metXH* | HSER(abcd) + THF(e) + SER(fgh) → MET(eabcd) + PYR(fgh) | n. | 60.0 |  |  | 2.3 | 1.5 | 0.1 |  | |
| *mez* | MAL(abcd) ↔ PYR(abd) + CO2(c) | n. | -1e-7 |  |  | >1e2 | <0.1 | nd |  | |
|  |  | x. | 5e-7 |  |  | - | <0.1 | - | [0, 5e5] | |
| *mqo_mdh* | MAL(abcd) ↔ OAA(abcd) | n. | 1801.1 |  |  | 97.3 | 44.1 | 2.5 |  | |
|  |  | x. | 25660.1 |  |  | - | 628.0 | - | [0, 5e5] | |
| *mu_mes* | BIO() ↔ BM() | n. | 0.41 |  |  | 0.02 | <0.1 | 0.00052 | [0.32, 0.48] | |
| *odh_1* | aKG(abcde) → SUC(abcd) + CO2(e) | n. | 894.1 |  |  | 91.1 | 21.9 | 2.3 |  | |
| *odh_2* | aKG(abcde) → SUC(badc) + CO2(e) | n. | 894.1 |  |  | 91.1 | 21.9 | 2.3 |  | |
| *pca_met_1* | KAP(abcdef) → SUC(baed) + ACoA(cf) | n. | 6.5 |  |  | 0.6 | 0.2 | 0.0 |  | |
| *pca_met_2* | KAP(abcdef) → SUC(baed) + ACoA(cf) | n. | 6.5 |  |  | 0.6 | 0.2 | 0.0 |  | |
| *pca_upt* | PCAin(abcdefg) → CO2(g) + KAP(abcdef) | n. | 13.0 |  |  | 1.3 | 0.3 | 0.0 | [0, 100] |  |
| *pck_ppc* | PEP(abc) + CO2(d) ↔ OAA(abdc) | n. | -43.9 |  |  | 99.5 | -1.1 | 2.4 |  | |
|  |  | x. | 1748.0 |  |  | 625.2 | 42.8 | 15.3 | [0, 5e5] | |
| *pdh* | PYR(abc) → ACoA(ab) + CO2(c) | n. | 3575.1 |  |  | 148.3 | 87.5 | 3.9 |  | |
| *pfk* | F6P(abcdef) → FBP(abcdef) | n. | 3264.2 |  |  | 115.3 | 79.9 | 3.1 |  | |
|  |  | x. | 1246.0 |  |  | 730.0 | 30.5 | 17.9 | [0, 5e6] | |
| *pgi* | G6P(abcdef) ↔ F6P(afbcde) | n. | 2711.0 |  |  | 309.1 | 66.4 | 7.6 |  | |
|  |  | x. | 95165.1 |  |  | - | 2329.1 | - | [0, 5e6] | |
| *pgm_ots* | G6P(abcdef) ↔ GXP(abcdef) | n. | 1 |  |  | - | 0.02 | - | - | |
|  |  | x. | 409.7 |  |  | - | 10.0 | - | [0, 5e6] | |
| *phoAB* | CHO(abcdefghij) → PHE(ghcdbafei) + CO2(j) | n. | 54.6 |  |  | 2.1 | 1.3 | 0.1 |  | |
| *proG* | GLU(abcde) → PRO(badce) | n. | 69.8 |  |  | 2.7 | 1.7 | 0.1 |  | |
| *pts* | PEP(abc) → PYR(abc) | n. | 4085.9 |  |  | 81.0 | 100.0 | 2.5 |  | |
| *pyc_odx* | PYR(abc) + CO2(d) ↔ OAA(abdc) | n. | 1325.5 |  |  | - | 32.4 | - | [-6001, 6134] | |
|  |  | x. | 5e-7 |  |  | - | <0.1 | - | [0, 5e5] | |
| *pyk* | PEP(abc) → PYR(abc) | n. | 1858.7 |  |  | - | 45.5 | - | [0, 6540] | |
| *sdh_1* | SUC(abcd) ↔ FUM(abcd) | n. | 900.6 |  |  | 91.1 | 22.0 | 2.3 |  | |
|  |  | x. | 42792.8 |  |  | - | 1047.3 | - | [0, 5e5] | |
| *sdh_2* | SUC(abcd) ↔ FUM(badc) | n. | 900.6 |  |  | 91.1 | 22.0 | 2.3 |  | |
|  |  | x. | 42792.8 |  |  | - | 1047.3 | - | [0, 5e5] | |
| *serC* | PGA(abc) → SER(abc) | n. | 336.5 |  |  | 13.0 | 8.2 | 0.3 |  | |
| *tal* | S7P(abcdefg) + GAP(hij) ↔ F6P(iajhec) + E4P(gbfd) | n. | 346.2 |  |  | 104.1 | 8.5 | 2.6 |  | |
|  |  | x. | 5e-7 |  |  | - | <0.1 | - | [0, 5e4] | |
| *thrB* | HSER(abcd) → THR(bacd) | n. | 196.0 |  |  | 7.6 | 4.8 | 0.2 |  | |
| *tkt1* | P5P(abcde) + P5P(fghij) ↔ S7P(agcifjh) + GAP(ebd) | n. | 346.2 |  |  | 104.1 | 8.5 | 2.6 |  | |
|  |  | x. | 6037.7 |  |  | 3143.5 | 147.8 | 77.0 | [0, 5e5] | |
| *tkt2* | E4P(abcd) + P5P(efghi) ↔ F6P(bedcag) + GAP(ifh) | n. | 236.1 |  |  | 105.6 | 5.8 | 2.6 |  | |
|  |  | x. | 535.7 |  |  | 150.1 | 13.1 | 3.7 | [0, 5e5] | |
| *tpi* | DHAP(abc) ↔ GAP(abc) | n. | 3264.2 |  |  | 115.3 | 79.9 | 3.1 |  | |
|  |  | x. | 12156.1 |  |  | 8764.6 | 297.5 | 214.6 | [0, 5e5] | |
| *tyrAB* | CHO(abcdefghij) → TYR(dbhcafgei) + CO2(j) | n. | 33.3 |  |  | 1.3 | 0.8 | 0.0 |  | |
| *acoa_bm* | ACoA(ab) → | n. | 1027.2 |  |  | 39.6 | 25.1 | 1.0 |  | |
| *akg_bm* | aKG(abcde) → | n. | 113.4 |  |  | 4.4 | 2.8 | 0.1 |  | |
| *ala_bm* | ALA(abc) → | n. | 249.0 |  |  | 9.6 | 6.1 | 0.3 |  | |
| *arg_bm* | ARG(abcdef) → | n. | 77.7 |  |  | 3.0 | 1.9 | 0.1 |  | |
| *asx_bm* | ASN(abcd) → | n. | 163.9 |  |  | 6.3 | 4.0 | 0.2 |  | |
| *bm_frac* | BM() → | n. | 0.41 |  |  | 0.02 | 0.01 | 0.00052 |  | |
| *co2_out* | CO2(a) → | n. | 8419.1 |  |  | 560.5 | 206.1 | 14.1 |  | |
| *cys_bm* | SER(abc) → | n. | 35.7 |  |  | 1.4 | 0.9 | 0.0 |  | |
| *e4p_bm* | E4P(abcd) → | n. | 22.2 |  |  | 0.9 | 0.5 | 0.0 |  | |
| *f6p_bm* | F6P(abcdef) → | n. | 29.2 |  |  | 1.1 | 0.7 | 0.0 |  | |
| *g6p_bm* | G6P(abcdef) → | n. | 84.2 |  |  | 3.2 | 2.1 | 0.1 |  | |
| *gap_bm* | GAP(abc) → | n. | 53.0 |  |  | 2.0 | 1.3 | 0.1 |  | |
| *gln_bm* | GLN(abcde) → | n. | 80.5 |  |  | 3.1 | 2.0 | 0.1 |  | |
| *glu_bm* | GLU(abcde) → | n. | 250.6 |  |  | 9.7 | 6.1 | 0.3 |  | |
| *gly_bm* | GLY(ab) → | n. | 148.3 |  |  | 5.7 | 3.6 | 0.2 |  | |
| *GXP_bm* | GXP(abcdef) → | n. | 1 |  |  | - | 0.02 | - |  | |
| *ile_bm* | ILE(abcdef) → | n. | 83.0 |  |  | 3.2 | 2.0 | 0.1 |  | |
| *leu_bm* | LEU(abcdef) → | n. | 180.8 |  |  | 7.0 | 4.4 | 0.2 |  | |
| *lys_bm* | LYS(abcdef) → | n. | 83.0 |  |  | 3.2 | 2.0 | 0.1 |  | |
| *met_bm* | MET(abcde) → | n. | 60.0 |  |  | 2.3 | 1.5 | 0.1 |  | |
| *oaa_bm* | OAA(abcd) → | n. | 199.7 |  |  | 7.7 | 4.9 | 0.2 |  | |
| *p5p_bm* | P5P(abcde) → | n. | 361.2 |  |  | 13.9 | 8.8 | 0.4 |  | |
| *pep_bm* | PEP(abc) → | n. | 43.6 |  |  | 1.7 | 1.1 | 0.0 |  | |
| *pga_bm* | PGA(abc) → | n. | 254.7 |  |  | 9.8 | 6.2 | 0.3 |  | |
| *phe_bm* | PHE(abcdefghi) → | n. | 54.6 |  |  | 2.1 | 1.3 | 0.1 |  | |
| *pro_bm* | PRO(abcde) → | n. | 69.8 |  |  | 2.7 | 1.7 | 0.1 |  | |
| *pyr_bm* | PYR(abc) → | n. | 94.1 |  |  | 3.6 | 2.3 | 0.1 |  | |
| *ser_bm* | SER(abc) → | n. | 92.4 |  |  | 3.6 | 2.3 | 0.1 |  | |
| *thf_out* | THF(a) → | n. | 88.3 |  |  | 3.4 | 2.2 | 0.1 |  | |
| *thr_bm* | THR(abcd) → | n. | 113.0 |  |  | 4.4 | 2.8 | 0.1 |  | |
| *tyr_bm* | TYR(abcdefghi) → | n. | 33.3 |  |  | 1.3 | 0.8 | 0.0 |  | |
| *val_bm* | VAL(abcde) → | n. | 116.7 |  |  | 4.5 | 2.9 | 0.1 |  | |

## Flux comparison


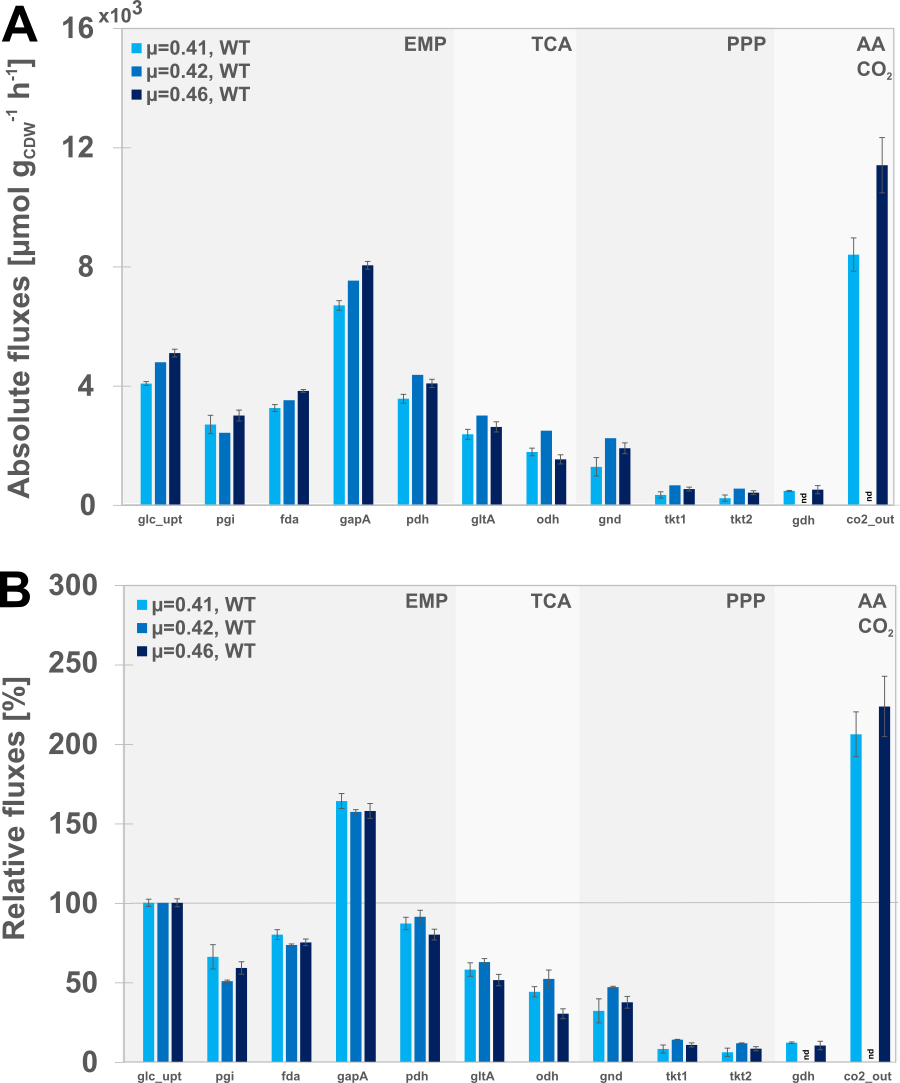


Supplementary Figure 5: Comparison of selected absolute (A) and relative (B) fluxes in µmol g_CDW_^-1^ h^-1^ and % uptGLC, respectively, obtained for *C. glutamicum* WT (ATCC 13032) cultivated in glucose minimal media at growth rates of µ = 0.41 h^-1^ (this study), µ = 0.42 h^-1^ [batch, shaking flask, label information derived from protein-bound amino acids, (Becker et al., 2011)], as well as µ = 0.46 h^-1^ [batch, shaking flask, label information derived from protein-bound amino acids (Wang et al., 2018)]. Absolute glucose uptake rates were 4,086 ± 65 µmol g_CDW_^-1^ h^-1^ for µ = 0.41 h^-1^_,_ 4,800 µmol g_CDW_^-1^ h^-1^ for µ = 0.42 h^-1^ and 5,110 ± 130 µmol g_CDW_^-1^ h^-1^ for µ = 0.46 h^-1^. Standard deviations of relative fluxes were calculated based on Gaussian error propagation. The study of Becker et al. (2011) assumed uptake rates to be error-free. The studies Wang et al. (2018), and Becker et al. (2011) relied on label information derived from protein-bound amino acids using GC-MS, whereas this work estimated fluxes from labeling information of intermediates and free amino acids derived with HILIC-Q-TOF-HRMS comprising free amino acids and central carbon intermediates. Since this assumption would yield overoptimistic flux standard deviations, these are not included in the figure. “nd” means that no flux values were available, AA amino acids.

# Bioprocess model used for external rate estimation

A mechanistic partially-structured model for batch and chemostat mode was adapted and simplified from (Cerff et al., 2013) to consistently describe the process data and estimate extracellular rates as well as their errors (standard deviations). The main output of the model, the rate estimates obtained for the chemostat phase, were then used for flux fitting. The model consists of three ODEs (Eqs. 1-3) for biomass concentration *c_x_*, glucose concentration *c_GLC_* and (accumulated) CO_2_ concentration *c_CO2_* (in g L^-1^). In chemostat mode, the reactor volume was constantly diluted by the rate *D* with fresh glucose solution, represented by *c_GLC,feed_*. The model has the general form:

$\frac{{dc}_{X}}{dt}=(\mu-D)\cdot c_{X}$ (Eq. 1)

$\frac{{dc}_{GLC}}{dt}={-q}_{GLC}\cdot c_{X}+D\cdot(c_{GLC,feed}-c_{GLC})$ (Eq. 2)

$\frac{{dc_{CO}}_{2}}{dt}={q_{CO}}_{2}\cdot c_{X}-D\cdot{c_{CO}}_{2}$ (Eq. 3)

The model considers the following intracellular processes: Glucose is utilized for anabolism and catabolism. Following anabolism, glucose is used for synthesis of biomass precursor molecules and biomass. In catabolism, glucose is metabolized to CO_2_ and synthesis of ATP.

Specific extracellular rates *q_GLC_*_,_ *q_CO2_* (in g g_CDW_^-1^ h^-1^) and *µ* (in h^-1^) and specific intracellular rates were derived by means of 5 analytic equations: Michaelis-Menten kinetics for glucose (Eq. 4), a stoichiometry linking anabolism (biomass formation, *µ*) and catabolism (CO_2_ formation, *q_CO2,_* (Eq. 5)) and three mass balances considering total glucose (Eq. 6), carbon in the anabolic branch (Eq. 7) and carbon in the catabolic branch (Eq. 8):

$q_{GLC}=q_{GLC,max}\cdot\frac{c_{GLC}}{c_{GLC}+K_{GLC}}$ (Eq. 4)

$q_{{CO}_{2}}=Y_{{CO}_{2},X}\cdot\mu$ (Eq. 5)

$q_{GLC}=q_{ana}+q_{cata}$ (Eq. 6)

$e_{C,GLC}\cdot q_{ana}=e_{C,X}\cdot\mu$ (Eq. 7)

$e_{C{,CO}_{2}}\cdot q_{{CO}_{2}}=e_{C,GLC}\cdot q_{cata}$ (Eq. 8)

To calculate the specific rates *q_i_*, the following model parameters were taken from the literature or calculated from physico-chemical constants: Carbon mass fraction of glucose *e_C,GLC_*= 0.4 (w/w), carbon mass fraction of the dry biomass *e_C,X_*= 0.5 (w/w) (Chmiel, 2011), carbon mass fraction of CO_2_ *e_C,CO2_*= 0.27 (w/w). The dilution rate *D = Q_GLC,feed_/V_R_* was determined with the constant working volume of the bioreactor *V_R_*= 1.2 L and the volumetric glucose feed rate of *Q_GLC,feed_*= 0.48 L h^-1^ in chemostat mode. The concentration of the feed was *c_GLC,feed_*= 11.6 g L^-1^.

The three parameters *q_GLC,max_* (max. glucose uptake rate), *K_GLC_* (Michaelis-Menten constant of glucose) and *Y_CO2,X_* (yield of CO_2_ per biomass) were estimated for batch and chemostat mode separately. Model parameters were fitted to in total 24 data points of extracellular glucose and biomass concentrations (supplementary figure 5A) assuming relative measurement errors of 5 %. For batch mode (0-7 h, *D*= 0 h^-1^), the following initial values are used:

- *c_x,0_* = 0.33 g L^-1^
- *c_GLC,0_* = 11.92 g L^-1^
- *c_CO2,0_*= 0 g L^-1^

Final concentration values from the batch simulation were used as initial conditions for the simulations of the chemostat mode (7-55.3 h, *D*= 0.4 h^-1^):

- *c_x,0_*= 6.12 g L^-1^
- *c_GLC,0_*= 1.8e-2 g L^-1^
- *c_CO2,0_*= 6.65 g L^-1^

For Monte Carlo simulations, 1,000 data sets were estimated independently for the parameters *q_GLC,max_*, *K_GLC_* and *Y_CO2,X_*. From each of those sets the corresponding set of biomass-specific rates (*µ*, *q_GLC_*, *q_CO2_*) were directly received, including their standard deviations. The maximum specific glucose uptake rate *q_GLC,max_* was only estimated for the batch mode and assumed to remain constant during chemostat mode. Offline CO_2_ production *q_CO2,exp_* was calculated based on fractions of CO_2_ in the exhaust air of the bioreactor according to:

$q_{CO2,exp}=\frac{\dot{V_{g}}\cdot M_{W,CO2}}{c_{X}{\cdot V}_{r}\cdot V_{g}}\cdot\left( y_{CO2,out}-y_{CO2,in}\cdot\frac{1-y_{O2,in}-y_{CO2,in}}{1-y_{O2,out}-y_{CO2,out}} \right)$ (Eq. 9)

$\dot{V_{g}}$ and *V_r_* are the gassing rate and the working volume of the bioreactor, *V_g_* is the specific volume of air at standard conditions (24.47 L mol^-1^). *M_W,CO2_* denotes the molecular weight of carbon dioxide, and the fractions *y_i_* [-] are the fractions of O_2_ and CO_2_ in the gassing inlet and outlet of the bioreactor. CO_2_-concentrations were not subject of the parameter fitting procedure.

The biomass coefficient of biomass on glucose was calculated by:

$Y_{X,GLC}=\frac{\mu}{q_{GLC}}$ (Eq. 10)

In order to receive an estimate for the molar PCA uptake rate, the equation

$\frac{{dc}_{PCA}}{dt}=-q_{PCA}\cdot c_{X}+D\cdot(c_{PCA,feed}-c_{PCA})$ (Eq. 11)

was applied to calculate *q_PCA_* under chemostat conditions (*dc_PCA_/dt*= 0) assuming constant values *D*= 0.4 h^-1^, *c_PCA,feed_*= 30 mg L^-1^, *c_PCA_*= 0 mg L^-1^, *c_x_* = 6 g L^-1^ and *M_w,PCA_*= 154.12 g mol^-1^.

All mass-related rates *q_i_* were transformed into molar-related rates *r_i_* for ^13^C-MFA according to:

$r_{i}=\frac{q_{i}}{M_{W,i}}$ (Eq. 12)

The molar weights are *M_W,GLC_*= 180 g mol^-1^, *M_W,CO2_*= 44 g mol^-1^, and *M_W,PCA_*= 154 g mol^-1^, respectively.


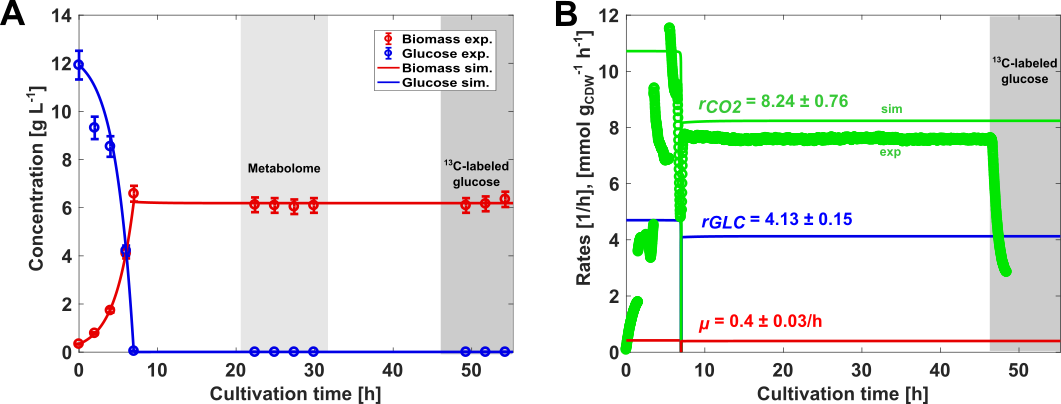


Supplementary Figure 6: Results from the bioprocess model. (A) Time course of offline biomass and glucose concentrations in g L^-1^. (B) Extracellular rates *r_CO2_*, *r_GLC_* (in mmol g_CDW_^-1^ h^-1^) and *µ* (in h^-1^) were estimated using the process model. The initial batch phase (0-7 h) was followed up by a chemostat mode (7-55.3 h). Metabolome samples were taken between 20-30 h cultivation time. Until 47 h of cultivation time, naturally labeled glucose was used, afterwards ^13^C-labeled D-glucose was applied until the end of cultivation. Circles denote offline measurements including calculated CO_2_ production rate from exhaust gas measurements. Continuous lines were simulated using the process model with fitted parameters: Batch: *Y_CO2,X_* = 1.12 ± 0.22g g_CDW_^-1^, *K_GLC_* = 24.4 ± 17.8 µmol L^-1^, *r_GLC,max_* = 4,701 ± 334 µmol g_CDW_^-1^ h^-1^. Chemostat: *Y_CO2,X_* = 0.91 ± 0.11 g g_CDW_^-1^, *K_GLC_* = 5.4 ± 2.5 µmol L^-1^, *r_GLC,max_* (see batch=const.). From these estimates specific rates were obtained, shown in (B). Furthermore, yield coefficients *Y_X,GLC_* = 0.50 ± 0.01 g_CDW_ g^-1^ (batch) and *Y_X,GLC_* = 0.53 ± 0.02 g_CDW_ g^-1^ (chemostat) were calculated.

Supplementary Table 3: Specific extracellular rates *r* (in µmol g_CDW_^-1^ h^-1^) and the growth rate *µ* (in h^-1^) estimated by non-linear regression with the model equations (Eq. 1)-(Eq. 9) and flux fitting. The order of magnitude of *r_PCA_* was estimated by analytically solving (Eq. 11) with a relative error of 10%.

| Rates | Process model  Batch | Process model  chemostat | | ^13^C-MFA |
| --- | --- | --- | --- | --- |
| *r_GLC_* | 4,699 ± 336 | 4,130 ± 150 | 4,086 ± 65 | |
| *r_GLC_* _,1-13C_ | - | 1,361 ± 49 | 1,395 ± 31 | |
| *r_GLC_* _,U-13C_ | - | 2,765 ± 100 | 2,691 ± 57 | |
| *r_CO2_* | 10,707 ± 2067 | 8,240 ± 760 | 8,419 ± 561 | |
| *r_PCA_* | - | 13.0 ± 1.0 | 13.0 ± 1.3 | |
| *µ* | 0.42 ± 0.01 | 0.40 ± 0.03 | 0.41 ± 0.02 | |

# References for supplementary material

Becker, J., Zelder, O., Stefan, H., Schr, H., and Wittmann, C. (2011). From zero to hero — Design-based systems metabolic engineering of *Corynebacterium glutamicum* for L-lysine production. 13, 159–168. doi:10.1016/j.ymben.2011.01.003.

Buchholz, J., Graf, M., Blombach, B., and Takors, R. (2014). Improving the carbon balance of fermentations by total carbon analyses. *Biochemical engineering journal*, 90, 162-169. doi: 10.1016/j.bej.2014.06.007

Cerff, M., Scholz, A., Käppler, T., Ottow, K. E., Hobley, T. J., and Posten, C. (2013). Semi-continuous in situ magnetic separation for enhanced extracellular protease production-modeling and experimental validation. *Biotechnol. Bioeng.* 110, 2161–72. doi:10.1002/bit.24893.

Chmiel, H. (2011). *Bioprozesstechnik*. Munich: Elsevier.

Feith, A., Teleki, A., Graf, M., Favilli, L., and Takors, R. (2019). Hilic-enabled ^13^C metabolomics strategies: Comparing quantitative precision and spectral accuracy of qtof high- and qqq low-resolution mass spectrometry. *Metabolites*. doi:10.3390/metabo9040063.

Kappelmann, J., Wiechert, W., and Noack, S. (2016). Cutting the Gordian Knot: Identifiability of anaplerotic reactions in *Corynebacterium glutamicum* by means of ^13^C-metabolic flux analysis. *Biotechnol. Bioeng.* 113, 661–674. doi:10.1002/bit.25833.

de Koning, W., and van Dam, K. (1992). A method for the determination of changes of glycolytic metabolites in yeast on a subsecond time scale using extraction at neutral pH. *Analytical biochemistry*, 204(1), 118-123.

Möllney, M., Wiechert, W., Kownatzki, D., and de Graaf, A. A. (1999). Bidirectional reaction steps in metabolic networks. IV. Optimal design of isotopomer labeling experiments. *Biotechnol. Bioeng.* 66, 86–103. doi:10.1002/(SICI)1097-0290(1999)66:2<86::AID-BIT2>3.0.CO;2-A.

Nöh, K., Niedenführ, S., Beyß, M., and Wiechert, W. (2018). A Pareto approach to resolve the conflict between information gain and experimental costs: Multiple-criteria design of carbon labeling experiments. *PLoS Comput. Biol.* 14, e1006533. doi:10.1371/journal.pcbi.1006533.

Teleki, A., Sánchez-Kopper, A., and Takors, R. (2015). Alkaline conditions in hydrophilic interaction liquid chromatography for intracellular metabolite quantification using tandem mass spectrometry. *Anal. Biochem.* 475, 4–13. doi:10.1016/j.ab.2015.01.002.

Teleki, A., Rahnert, M., Bungart, O., Gann, B., Ochrombel, I., and Takors, R. (2017). Robust identification of metabolic control for microbial l-methionine production following an easy-to-use puristic approach. Metabolic engineering, 41, 159-172. doi: 10.1016/j.ymben.2017.03.008

Wang, Z., Liu, J., Chen, L., Zeng, A., Solem, C., and Jensen, P. R. (2018). Alterations in the transcription factors GntR1 and RamA enhance the growth and central metabolism of *Corynebacterium glutamicum*. *Metab. Eng.* 48, 1–12. doi:10.1016/j.ymben.2018.05.004.
